# Management of pharmaceutical and recreational drug poisoning

**DOI:** 10.1186/s13613-020-00762-9

**Published:** 2020-11-23

**Authors:** Bruno Mégarbane, Mathieu Oberlin, Jean-Claude Alvarez, Frederic Balen, Sébastien Beaune, Régis Bédry, Anthony Chauvin, Isabelle Claudet, Vincent Danel, Guillaume Debaty, Arnaud Delahaye, Nicolas Deye, Jean-Michel Gaulier, Francis Grossenbacher, Philippe Hantson, Frédéric Jacobs, Karim Jaffal, Magali Labadie, Laurence Labat, Jérôme Langrand, Frédéric Lapostolle, Philippe Le Conte, Maxime Maignan, Patrick Nisse, Philippe Sauder, Christine Tournoud, Dominique Vodovar, Sebastian Voicu, Pierre-Géraud Claret, Charles Cerf

**Affiliations:** 1 Department of Medical and Toxicological Critical Care, Federation of Toxicology, Lariboisière Hospital, AP-HP, INSERM MURS-1144, University of Paris, 2 Rue Ambroise Paré, Paris, 75010 France; 2 Emergency Department, HuManiS Laboratory (EA7308), University Hospital, Strasbourg, France; 3 Department of Pharmacology and Toxicology, Inserm U-1173, FHU Sepsis, Raymond Poincaré Hospital, AP-HP, Paris-Saclay University, Garches, France; 4 Emergency Department, Toulouse University Hospital, Toulouse, France; 5 Department of Emergency Medicine, Ambroise Paré Hospital, AP-HP, INSERM UMRS-1144, Paris-Saclay University, Boulogne-Billancourt, France; 6 Hospital Secure Unit, Pellegrin University Hospital, Bordeaux, France; 7 Emergency Department, Hôpital Lariboisière, AP-HP, Paris, France; 8 Pediatric Emergency Department Children’s Hospital CHU Toulouse, Toulouse, France; 9 Department of Emergency Medicine, University Hospital of Grenoble, Grenoble, France; 10 5525, University Grenoble Alps/CNRS/CHU de Grenoble Alpes/TIMC-IMAG UMR, Grenoble, France; 11 Intensive Care Unit, Rodez Hospital, Rodez, France; 12 Department of Medical and Toxicological Critical Care, Federation of Toxicology, Lariboisière Hospital, AP-HP, INSERM U942, University of Paris, Paris, France; 13 Laboratory of Toxicology, EA 4483 - IMPECS - IMPact de L’Environnement Chimique Sur La Santé Humaine, University of Lille, Lille, France; 14 Emergency Department, Reims University Hospital, Reims, France; 15 Intensive Care Department, Cliniques Universitaires St-Luc, Brussels, Belgium; 16 Polyvalent Intensive Care Unit, Antoine Béclère Hospital, Assistance Publique-Hôpitaux de Paris, Paris-Sud University, Clamart, France; 17 Poison Control Centre of Bordeaux, University Hospital of Bordeaux, Bordeaux, France; 18 Laboratory of Toxicology, Federation of Toxicology APHP, Lariboisière Hospital, INSERM UMRS-1144, University of Paris, Paris, France; 19 Poison Control Center of Paris, Federation of Toxicology, Fernand-Widal-Lariboisière Hospital, AP-HP, INSERM UMRS-1144, University of Paris, Paris, France; 20 SAMU 93-UF Recherche-Enseignement-Qualité, Inserm, U942, Avicenne Hospital, AP-HP, Paris-13 University, Bobigny, France; 21 Department of Emergency Medicine, University Hospital of Nantes, Nantes, France; 22 Emergency Department, Grenoble University Hospital, INSERM U1042, Grenoble Alpes University, Grenoble, France; 23 Poison Control Centre, University Hospital of Lille, Lille, France; 24 Intensive Care Unit, University Hospital of Strasbourg, Strasbourg, France; 25 Poison Control Centre, University Hospital of Nancy, Nancy, France; 26 Department of Anesthesia Resuscitation Pain Emergency Medicine, Nîmes University Hospital, Nîmes, France; 27 Intensive Care Unit, Foch Hospital, Suresnes, France

**Keywords:** Guidelines, Poisoning, Intoxication, Pharmaceutical drug, Recreational drug, Antidote

## Abstract

**Background:**

Poisoning is one of the leading causes of admission to the emergency department and intensive care unit. A large number of epidemiological changes have occurred over the last years such as the exponential growth of new synthetic psychoactive substances. Major progress has also been made in analytical screening and assays, enabling the clinicians to rapidly obtain a definite diagnosis.

**Methods:**

A committee composed of 30 experts from five scientific societies, the *Société de Réanimation de Langue Française* (SRLF), the *Société Française de Médecine d’Urgence* (SFMU), the *Société de Toxicologie Clinique* (STC), the *Société Française de Toxicologie Analytique* (SFTA) and the *Groupe Francophone de Réanimation et d’Urgences Pédiatriques* (GFRUP) evaluated eight fields: (1) severity assessment and initial triage; (2) diagnostic approach and role of toxicological analyses; (3) supportive care; (4) decontamination; (5) elimination enhancement; (6) place of antidotes; (7) specificities related to recreational drug poisoning; and (8) characteristics of cardiotoxicant poisoning. Population, Intervention, Comparison, and Outcome (PICO) questions were reviewed and updated as needed, and evidence profiles were generated. Analysis of the literature and formulation of recommendations were then conducted according to the GRADE^®^ methodology.

**Results:**

The SRLF-SFMU guideline panel provided 41 statements concerning the management of pharmaceutical and recreational drug poisoning. Ethanol and chemical poisoning were excluded from the scope of these recommendations. After two rounds of discussion and various amendments, a strong consensus was reached for all recommendations. Six of these recommendations had a high level of evidence (GRADE 1±) and six had a low level of evidence (GRADE 2±). Twenty-nine recommendations were in the form of expert opinion recommendations due to the low evidences in the literature.

**Conclusions:**

The experts reached a substantial consensus for several strong recommendations for optimal management of pharmaceutical and recreational drug poisoning, mainly regarding the conditions and effectiveness of naloxone and *N*-acetylcystein as antidotes to treat opioid and acetaminophen poisoning, respectively.

## Background

Poisoning is probably one of the leading causes of admission to emergency departments and intensive care units. A large number of radical epidemiological changes have occurred over the last decade, particularly the US opioid overdose crisis and the exponential growth of new synthetic recreational drugs called "new psychoactive substances" (NPS). Major progress has also been made in the field of analytical screening and assay techniques, now enabling the physicians in charge of the poisoned patient to increasingly rapidly obtain a definite diagnosis.

The *Société de Réanimation de Langue Française* (SRLF) and the *Société Française de Médecine d’Urgence* (SFMU), with the participation of the *Société de Toxicologie Clinique* (STC), the *Société Française de Toxicologie Analytique* (SFTA) and the *Groupe Francophone de Réanimation et d’Urgences Pédiatriques* (GFRUP) decided to revise the 2005 clinical practice guidelines on acute poisoning. The objective of these guidelines, based on analysis of the level of evidence in the literature, was to clarify the diagnostic approach, patient triage and therapeutic management. Because of the very wide range of toxins potentially involved, it was decided to focus these guidelines on pharmaceutical and recreational drug poisoning, excluding ethanol and chemical poisoning. These guidelines were also designed to cover all emergency care settings, from the pre-hospital setting (telephone triage by SAMU emergency services and on-site intervention by a doctor or SMUR mobile emergency and intensive care unit) to the hospital emergency department and intensive care unit. No specific guidelines for children were adopted, but any paediatric specificities of clinical presentation or management were identified in each recommendation.

There is a real risk of exposure to a xenobiotic (or a substance that is foreign to the human body), whether intentional or accidental, in modern society, which can sometimes lead to serious or even fatal poisoning. We will define "*exposure*" by contact with a xenobiotic, regardless of the route and "*poisoning*" by the presence of clinical (somatic and/or mental) manifestations, or laboratory and/or electrocardiographic abnormalities resulting from this exposure. We will define the "*reported dose*" of exposure as that reported by the patient during clinical interview or that reported by the entourage or first responders according to the signs observed at the time of discovery of the patient (empty blister packs, for example) and the "*potentially toxic dose*" as the dose that can theoretically lead to the onset of toxic signs, for example a supratherapeutic dose of a medication. The guidelines have strived to distinguish functional toxins from so-called "organ-damaging" toxins, to guide the clinical reasoning, prognostic approach and prioritization of dosage and type of treatment. As a reminder, a toxin is said to be *functional* when it transiently interferes with the function of an organ and the severity and outcome of poisoning induced by a functional toxin depend on its concentration in the target organ. A toxin is said to be "organ-damaging" when it causes organ damage, the severity of which depends on the maximum concentration in this target organ, while the outcome is independent of plasma concentrations, with a risk of disorders that can persist despite elimination of the toxin. The guidelines also address the four usual aspects of treatment that need to be considered in any patient, in whom poisoning is confirmed or suspected: supportive care, antidotes (or specific treatments), decontamination (i.e. designed to reduce the bioavailability of the toxin) and elimination enhancing treatments (i.e. designed to enhance elimination of the toxin that has already entered the internal environment). The concept of antidote was considered from a restrictive point of view, limited to drugs that have been clearly established to act on toxicokinetics or toxicodynamics, allowing improvement of the poisoned patient's functional or vital prognosis.

Finally, these guidelines provided an opportunity to highlight the role *of poison control and toxicovigilance centres* and the important role played by expert centres. Poison control centres are centres that provide information about toxic risks to health care professionals and the general public, as well as telephone assistance in the diagnosis, management and treatment of poisoning, with an active role in toxicovigilance. *Expert centres* are emergency or intensive care facilities with more extensive experience of acute toxicology, with access to more rarely used antidotes and/or able to rapidly display exceptional treatments. Of note, extrapolation of these guidelines, adapted first to the French practice, to other countries should be adjusted to the local situations such as travelling times to the hospital when considering the recommendations on pre-hospital interventions.

## Methods

These guidelines are the result of the work conducted by an SRLF and SFMU joint expert committee. The expert committee agenda was defined at the beginning of the study. The organizing committee initially defined the questions to be addressed in collaboration with the coordinators and then appointed experts in charge of each question. Questions were formulated according to a Patient Intervention Comparison Outcome (PICO) format after the first expert committee meeting. Review of the literature and formulation of recommendations were then conducted according to the Grade of Recommendation Assessment, Development and Evaluation (GRADE) methodology.

A level of evidence was defined for each publication cited as a function of the study design. This level of evidence could be revised by taking into account the methodological quality of the study. A global level of evidence was determined for each endpoint by taking into account the levels of evidence of each publication, the consistency of the results between the various studies, the direct or indirect nature of the evidence, and the cost analysis (Table 1). A "high" global level of evidence permitted the formulation of a "strong" recommendation (it is recommended to… GRADE 1+ or it is not recommended to GRADE 1−). When the global level of evidence was moderate, low or very low, an optional recommendation was formulated (it is probably recommended to… GRADE 2+ it is probably not recommended to… GRADE 2−). When the literature was non-existent or insufficient, the recommendation concerning the question was based on expert opinion (the experts suggest…). Proposed recommendations were presented and discussed one by one. The purpose of this process was not to inevitably reach a unique, convergent expert consensus on all of the proposals, but to define points of concordance, divergence or indecision. Each recommendation was then evaluated by each of the experts, who provided an individual score using a scale ranging from 1 (complete disagreement) to 9 (complete agreement). The collective score was established according to a GRADE grid methodology. In order to validate a recommendation according to a particular criterion, at least 50% of experts had to express an opinion globally in favour of the recommendation, while less than 20% of experts expressed an opposite opinion.

**Table 1:**
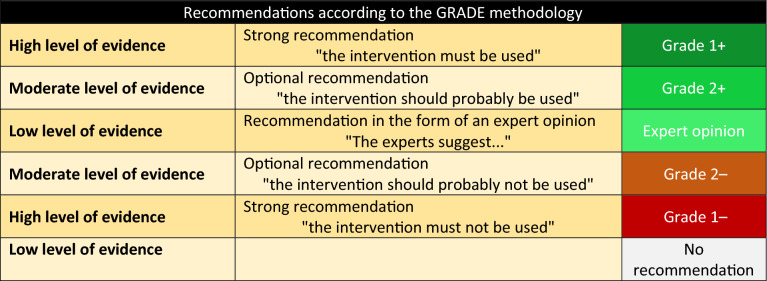
Recommendations according to the GRADE methodology

To obtain a strong recommendation, 70% of experts had to agree with the recommendation. In the absence of a strong consensus, the recommendations were reformulated and rescored in order to reach a consensus. Only expert opinions that obtained a strong consensus were finally adopted.

Scope of recommendations: eight fields were defined: (1) severity assessment and initial triage; (2) diagnostic approach and role of toxicological analyses; (3) supportive care; (4) decontamination; (5) elimination enhancement; (6) place of antidotes; (7) specificities related to recreational drug poisoning; and (8) characteristics of cardiotoxicant poisoning. A literature search was conducted on the PubMed and Cochrane Medline databases. To be included in the analysis, publications had to be written in French or English. The literature review focused on recent data according to an order of assessment ranging from meta-analyses and randomized trials to observational studies.

Summary of the results: Analysis of the literature by the experts and application of the GRADE methodology resulted in 42 recommendations. Six of the 42 formal recommendations had a high level of evidence (GRADE 1/−) and 6 had a low level of evidence (GRADE 2/−). The GRADE methodology could not be applied to 30 recommendations, resulting in an expert opinion. After two scoring rounds and amendments, a strong consensus was reached for all recommendations.

### Field 1: Severity assessment and initial triage

#### Question 1.1: Should a specific score be used to predict severity in a patient with suspected pharmaceutical or recreational drug poisoning?

STRONG RECOMMENDATION/GRADE 1−/STRONG CONSENSUS

**R 1.1: The Épidémiologie Toxicologie Clinique (ETC), Medical Priority Dispatch System (MPDS) and Poisoning Severity Score (PSS) scores should not be used at the time of telephone triage and at the first pre-hospital and hospital medical contact to assess the severity of pharmaceutical or recreational drug poisoning.**

*Rationale* At the time of triage of a telephone call for poisoning, use of the ETC score [1] or "Medical Priority Dispatch System (MPDS)" triage system is not recommended due to overestimation (ETC) or underestimation (PDS) of the poisoning severity [2]. A consciousness rating scale (Glasgow score, Alert Verbal Pain Unresponsive scale), assessed by a trained first responder, can be useful [3, 4]. In the pre-hospital setting and emergency departments, no multipurpose severity score [Simplified Acute Physiology Score (IGS or SAPS), Sequential Organ Failure Assessment (SOFA), Acute Physiology and Chronic Health Evaluation (APACHE)] has been shown to have a sufficient predictive value to allow early, individual detection of the risk of complications, the need for intensive care unit admission or death [1]. The complexity, the poor inter-individual reproducibility and the lack of validation of the Poisoning Severity Score (PSS) strongly limit its use in routine clinical practice. The development of a highly reliable poisoning severity score would appear to be utopian. The variability of expected toxic effects, for example between benzodiazepine poisoning and calcium-channel blocker poisoning, limits the generalization of severity scores. Furthermore, the risk associated with certain specific and less common forms of poisoning (chloroquine and metformin, for example) would be difficult to assess with a global severity score. At the present time, there is no clinical decision rule that can be used to confirm the benign nature of poisoning.

#### Question 1.2: At the time of telephone triage, in a patient with suspected pharmaceutical or recreational drug poisoning, what criteria should be used to dispatch a medical emergency team?

RECOMMENDATION IN THE FORM OF AN EXPERT OPINION/STRONG CONSENSUS

**R 1.2: In the pre-hospital setting, in a patient with suspected pharmaceutical or recreational drug poisoning, the experts suggest dispatching a medical emergency team in the presence of neurological, haemodynamic or respiratory failure. In other cases, the risk of rapid deterioration as a function of the clinical setting, time since exposure and the need for possible rapid treatment must be taken into account when deciding on the need for pre-hospital medical intervention.**

*Rationale* No studies with a high level of evidence have assessed the risk factors indicating the need for pre-hospital medical intervention of patients with suspected pharmaceutical or recreational drug poisoning. Only one French observational study [5] has evaluated the organization of pre-hospital management of poisoning. The authors compared the initial pre-hospital triage of poisoning patients [emergency room (ER), continuing care unit (CCU) or intensive care unit (ICU)] with the final hospital referral (ER, CCU or ICU) in a total of 2227 poisoning patients. Overestimating the patient's severity was associated with a lack of available toxicological information and, to a lesser extent, younger age. Underestimation of severity was associated with ingestion of antipsychotic, anticonvulsant and cardiovascular drugs. Observational studies and case reports have concluded that pre-hospital medical intervention may be useful for poisoning patients requiring an injection of antidote or orotracheal intubation, for example [6–10]. English-language studies have reported that early invasive pre-hospital intervention may reduce the morbidity and mortality of poisoned patients [6, 11–13].

#### Question 1.3: What are the criteria for admission to ICU, CCU and/or expert centre in a patient with suspected pharmaceutical or recreational drug poisoning managed in an emergency department (SMUR or emergency room)?

RECOMMENDATION IN THE FORM OF AN EXPERT OPINION/STRONG CONSENSUS

**R 1.3.1: For patients managed in an emergency department, the experts propose ICU admission in the presence of:**

- documented organ failure (clinical, laboratory, or electrocardiographic (ECG) signs) requiring close monitoring and/or specific management;
- exposure to any cardiotoxic pharmaceutical or substance in the presence of any abnormal objective signs (clinical, laboratory or ECG);
- exposure to any supposedly toxic pharmaceutical or recreational drug that can induce organ failure (neurological, cardiovascular and/or respiratory), even in patients with few or no symptoms managed within 6 h after the reported exposure (or longer, for extended-release forms).

RECOMMENDATION IN THE FORM OF AN EXPERT OPINION/STRONG CONSENSUS

**R 1.3.2: The experts suggest that admission to an expert centre should be proposed immediately in the case of poisoning possibly requiring the use of limited-access therapy (e.g. extracorporeal membrane oxygenation (ECMO), a specific enhanced elimination technique or a limited availability antidote).**

*Rationale* The indication for admission to a critical care unit (ICU, CCU, or even an expert centre) is based on clinical (toxidromes) and ECG signs, but also on the toxic potential related to the nature of the involved toxin, the reported ingested dose and time of exposure, as well as the patient's clinical background [14–16]. The most common toxins requiring ICU admission are cardiovascular drugs, almost systematically, and psychotropic drugs associated with a risk of serious complications such as tricyclic antidepressants or antipsychotics [17–19]. The onset of organ failure, including respiratory, neurological or haemodynamic failure, requires admission to an ICU [20]. Indications for renal replacement therapy (RRT), sometimes based on levels of certain plasma parameters, have been defined for certain serious poisonings [21–23].

A patient with suspected toxic shock or poisoning with substances such as cardiotoxic agents known to be responsible for severe toxicity, requires close monitoring, or even a rapid, aggressive, multimodal therapeutic approach in the presence of symptoms. Transfer to an expert centre equipped with extracorporeal life support (ECMO) should be considered for a patient in shock requiring the use of increasing doses of catecholamines. In the absence of response of the shock to conventional therapies or persistent cardiac arrest of toxic or presumed toxic origin, several studies have suggested that the use of ECMO improves the prognosis [24, 25].

#### Question 1.4: In a patient with pharmaceutical or recreational drug poisoning, who has undergone an initial somatic medical assessment, what are the clinical and/or complementary criteria justifying management outside a unit equipped with somatic medical monitoring?

RECOMMENDATION IN THE FORM OF AN EXPERT OPINION/STRONG CONSENSUS

**R 1.4: The experts suggest that asymptomatic patients following suspected pharmaceutical or recreational drug exposure can be managed in a unit without medical monitoring when the toxins have been clearly identified, when they have a short half-life, when any additional investigation justified by the properties of the toxin (including laboratory tests and ECG) are normal and when an initial psychiatric assessment has been carried out in case of attempted suicide.**

*Rationale* A significant proportion of patients admitted to the ER for pharmaceutical or recreational drug poisoning do not require somatic monitoring in a short-stay unit. Such patients represented 83% of poisoned patients in a Scottish prospective study [26], and 42% in a retrospective UK study [27]. Acetaminophen was the molecule most commonly involved in these poisonings (39% and 42%, respectively). In two French [17] and Belgian [28] studies, benzodiazepines were involved in 78% and 51% of cases, respectively. No cases of return home were reported, but 30% of patients were directly transferred to a psychiatric unit [17]. No complications were reported among non-admitted patients. Non-admission to a somatic hospital unit therefore appears possible and safe after systematic clinical evaluation, possibly associated with complementary examinations (ECG, laboratory work-up including serum acetaminophen concentrations, when indicated) to ensure that the following conditions are met: (1) well identified toxin(s), not causing an serious effects or organ damage, short half-life and (2) normal physical examination, notably normal vital parameters, conscious and oriented patient and no residual psychoactive effect. In the case of attempted suicide, an initial psychiatric assessment must be carried out in the emergency room before discharging the patient home or before admission to a psychiatric unit.

### Field 2: Diagnostic approach and the place of toxicology analyses

#### Question 2.1: Does a call to a poison control centre (PCC) or expert centre improve the management of a patient with suspected pharmaceutical or recreational drug poisoning?

RECOMMENDATION IN THE FORM OF AN EXPERT OPINION/STRONG CONSENSUS

**R 2.1: The experts suggest that a PCC and/or expert centre opinion should be obtained in cases of particularly severe or complex pharmaceutical or recreational drug poisoning.**

*Rationale* No published study of sufficient quality has provided conclusive evidence for the contribution of PCC and expert centres to the improvement of the management of patients with pharmaceutical or recreational drug poisoning, whether in terms of toxin identification or expected morbidity and mortality. Note that, in terms of identification of the suspected toxin: (1) due to the detailed knowledge of clinical toxicology (toxidromes), these expert centres can help to identify the class of toxins consumed [29]; (2) as in other countries [30], the dedicated and trained pharmacists of PCCs are able to identify medication tablets marketed in France 24 h a day, 7 days a week; and (3) PCC/expert centres work in collaboration with laboratories able to identify the possible presence of a toxic substance (tablet or liquid) within a package. In terms of morbidity and mortality, it should be noted that: (1) consultation of a PCC could reduce the length of hospital stay (probably by recommending earlier discharge from hospital than that envisaged in the absence of PCC opinion) [31–33]; and (2) PCC/expert centres can rapidly guide clinicians concerning the indications for antidotes, toxin elimination methods and the indications for exceptional techniques (ECMO). More extensive data are available concerning the role of PCCs in the utilization of healthcare facilities. Several studies have shown that PCC consultation can avoid admission to the emergency room or to the hospital and unnecessary complementary investigations when the PCC is contacted at the pre-hospital stage, by doctors or directly by the public [32, 34–38]. In addition, early contact with the PCC/expert centre may allow early referral of a patient to the most appropriate healthcare facility (ER, ICU, availability of an antidote/dialysis/ECMO, etc.). All of these studies suggest that consultation with a PCC and/or expert centre is a useful approach to optimize management of complex or particularly severe poisoning cases and to reduce costs because more appropriate resources and therapies are employed.

#### Question 2.2: Does routine screening for the main toxins improve the management of patients with suspected pharmaceutical or recreational drug poisoning?

RECOMMENDATION IN THE FORM OF AN EXPERT OPINION/STRONG CONSENSUS

**R 2.2: The experts suggest that routine screening should not be performed in order to improve the management of patients with suspected pharmaceutical or recreational drug poisoning. However, screening can be performed for information purposes.**

*Rationale* No published study with a good level of evidence has assessed the contribution of routine screening in patients with suspected poisoning. In a patient with suspected poisoning, the experts recommend a clinical approach based on clinical features (toxidromes) rather than on the non-quantitative results of blood or urine toxicology screening tests. Screening tests are not sufficient to establish a diagnosis or prognosis, or to monitor the kinetics of one or more toxins and their metabolites [39–41]. Urinary screening provides complementary information to blood screening, over a larger screening window, but the results of urine screening can never be used to interpret the toxidrome observed at the time of the urine sample.

Screening can be useful in specific situations: when the clinical diagnosis has not been established, complementary examinations are incompatible with the patient's history or in the presence of circulatory failure or unexplained coma. However, any toxicological screening tests must be systematically completed by targeted blood toxicology screening in order to assay blood concentrations, which are more closely correlated with toxicity [42, 43]. In order to more precisely document certain cases, it may be useful to take blood and urine samples at admission, and possibly repeat these samples during the toxin elimination in hospital. A biological sample collection (serum/plasma or urine samples) should always be considered at the time of the patient's admission when the aetiology is unclear or in the presence of signs of severity [44].

Two types of screening methods are recommended (Table 2): (1) rapid response methods (immunological and enzymatic), mainly for substances only detected in urine. These methods are of little value for screening of drug classes, due to their lack of specificity and sensitivity. (2) Methods that provide a response in less than 24 h, based on specialized techniques (liquid or gas chromatography), using various types of mass spectrometry (MS) and/or diode array detection [45, 46]. Semiquantitative blood screening can be a useful diagnostic tool in the same way as specific drug assays. The recent development of high-resolution MS technologies represents a real technological progress, allowing non-targeted screening methods, the only available technical solution at the present time to allow identification of unknown structures such as NPS.

**Table 2:** Toxicological screening

Toxicological screening methods			Comments–interpretation
Rapid screening methods on an automated chemistry analyzer	Immunological and enzymatic	Urine screens for illicit drugs and/or their metabolites (cocaine, amphetamines, opiates, etc.) without assay	Useful for “conventional” drugs, excluding NPS
			These tests need to be carefully interpreted (molecule identified by antibody, toxicokinetics, screening window, interferences, etc.)
			Limits of interpretation on urine
		Specific drug or toxin screens with blood and/or urine assays	Useful for blood assays (drugs, ethanol, etc.)
			Limits of interpretation on urine
		Drug class screens (benzodiazepines, tricyclic antidepressants, etc.) in blood and/or urine	Limits of interpretation for a drug of the class identified by antibody due to cross-reactions with other drugs of the same class and possible interferences
			Need for biological interpretation with respect to the toxicity thresholds of each drug
			Limits of interpretation for urine
Chromatographic confirmation of screening methods	Liquid or gas chromatography	Detection by diode arrays and/or mass spectrometry in blood and/or urine	Useful for broader targeted screening (up to 1200 molecules and/or metabolites)
			Need for biological interpretation of the nature of the molecules identified, the level of screening (sensitivity) and interpretation with respect to reported toxic concentrations
			A semiquantitative approach can be used simultaneously with screening for certain molecules
			Limits of interpretation on urine
	Liquid chromatography	High-resolution mass spectrometry (HRMS)	Useful to identify unknown chemical structures (non-targeted screening) (e.g. NPS)

#### Question 2.3: Does assay of reported or identified molecules improve the management of patients with suspected pharmaceutical or recreational drug poisoning?

STRONG RECOMMENDATION/GRADE 1+/STRONG CONSENSUS

**R 2.3.1: Drug assays must be performed when the suspected ingested molecules are acetaminophen, acetylsalicylic acid, valproic acid, digoxin and lithium.**

RECOMMENDATION IN THE FORM OF AN EXPERT OPINION/STRONG CONSENSUS

**R 2.3.2: The experts suggest that plasma or serum assays of the suspected ingested molecules should be performed in the presence of complex or particularly severe clinical settings, when recommended by an expert centre.**

*Rationale* The assay of certain drugs may optimize the patient management by indicating the need for RRT or the use of a specific antidote, the application or dose of which may be concentration-dependent. Drug assays in acute poisonings are only useful when performed early after exposure and in serum/plasma (Table 3). These assays are fully automated for acetaminophen, salicylates, valproic acid, digoxin and now also for lithium (specific electrodes). The results can therefore be obtained within 120 min [47].

**Table 3:** Toxicological assays

Dosing methods			Interest/limits
Immunological	Plasma or serum	Acetaminophen, salicylic acid (active metabolite of acetylsalicylic acid), digoxin, valproic acid	Automated assays (available in less than 120 min) Few potential interferences (kit-dependent)
Specific electrodes	Plasma (without lithium heparin as anticoagulant) or serum	Lithium	
LC–MS/MS or GC–MS	Plasma or serum	Acetaminophen, salicylic acid (active metabolite of acetylsalicylic acid), digoxin, valproic acid	No interference Not automated, cannot be used in an emergency
Flame photometer	Plasma or total blood (erythrocyte assay)	Lithium	

*LC–MS/MS* liquid chromatography coupled with tandem mass spectrometry, *GC–MS* gas chromatography coupled with mass spectrometry

RRT is recommended for valproic acid poisoning in the presence of a serum concentration greater than 1300 mg/L (or even 900 mg/L, with discontinuation of RRT as soon as the valproic acid levels decrease to between 50 and 100 mg/L) [48]. In cases of lithium poisoning, depending on the type of poisoning (acute versus acute on chronic versus chronic), the severity of neurological features and the degree of renal impairment, serum lithium levels can be used to determine the indications for RRT. RRT is systematically recommended for serum lithium concentrations greater than 4 mmol/L in combination with clinical signs of severity or even systematically when serum lithium concentrations are greater than 5 mmol/L [21]. In cases of salicylate poisoning, serum salicylate levels > 1000 mg/L by H6 require the use of RRT, regardless of the clinical features [22].

For acetaminophen poisoning with a reported ingested dose ≥ 8 g (250 mg/kg before the age of 6 years, and 150 mg/kg after the age of 6 years) and in the absence of repeated intake, *N*-acetylcysteine administration is guided by interpretation of plasma acetaminophen concentrations as a function of time since exposure, according to the Rumack and Matthew nomogram [49]. When *N*-acetylcysteine is not available and in the presence of extremely high plasma acetaminophen levels (greater than 1000 mg/L or 700 mg/L in the presence of signs of mitochondrial dysfunction), RRT may be considered, as acetaminophen is dialysable [50]. Finally, in cases of acute or chronic digoxin poisoning, documented by plasma digoxin levels greater than 2.6 nmol/L and in the presence of compatible clinical features, plasma digoxin levels can be used to calculate the quantity of digoxin antibody Fab fragments to be administered (for molar or semimolar neutralization) [51, 52]. Of note, screening for acetaminophen would also be useful in patients that are either unconscious or unreliable (e.g. genuine death wish).

### Field 3: Symptomatic treatment

#### Question 3.1: What are the criteria for endotracheal intubation in a patient with pharmaceutical or recreational drug poisoning?

RECOMMENDATION IN THE FORM OF AN EXPERT OPINION/STRONG CONSENSUS

**R 3.1: In the presence of haemodynamic, neurological or respiratory failure (not reversible by antidote), the experts suggest that endotracheal intubation should be performed with rapid sequence induction.**

*Rationale* No published study with a good level of evidence is available concerning the indications for endotracheal intubation in patients with pharmaceutical or recreational drug poisoning. In the observational studies, the majority of cases of poisoning associated with intubation involved hypnotics, antidepressants and opioids [53, 54]. By analogy with the proposed management of head injury, a Glasgow Coma Score less than 8 is often used as an indication for intubation [4, 55, 56]. However, no published study is available to support this indication [57]. The Glasgow Coma Score does not predict loss of the swallowing [58] or cough reflexes [59]. A Glasgow score greater than 8 in the poisoned patients also does not rule out the possibility of aspiration pneumonia, the risk of which appears to increase with decreasing level of consciousness in several observational studies [60, 61]. Other factors also appear to be associated with the risk of aspiration, such as patient positioning [62], the type of toxin [61], the use of gastric lavage and administration of activated charcoal, whether or not the patient is intubated [63]. Withholding intubation in patients with a Glasgow Coma Score < 8 such as in γ-hydroxybutyric acid-intoxicated patients is possible based on a case-by-case analysis of patient’s clinical conditions and the estimated elimination half-live of the involved toxicant.

Apart from bradypnea in the context of opioid poisoning, no observational study has evaluated intubation of patients with signs of respiratory distress. According to case reviews based on small series of salicylate poisoning, respiratory alkalosis is abolished by intubation, which is responsible for an increased incidence of acidaemia [64].

When intubation is decided, rapid sequence induction is associated with a lower rate of difficult intubations and mortality in the poisoned patients, regardless of their level of consciousness [54, 65, 66].

#### Question 3.2: Should a haemodynamic assessment be performed in a patient with pharmaceutical or recreational drug poisoning in shock and, if so, how should it be performed?

RECOMMENDATION IN THE FORM OF AN EXPERT OPINION/STRONG CONSENSUS

**R 3.2: In a patient with pharmaceutical or recreational drug poisoning in shock, the experts suggest that a haemodynamic assessment should be performed in parallel with the clinical and laboratory evaluation, and should be repeated according to the subsequent clinical course. The experts recommend the same modalities as those recommended for assessment of non-toxic shock.**

*Rationale* No randomized clinical trial has compared the impact of haemodynamic assessment on morbidity and mortality in the poisoned patients. No observational study, with or without propensity score, and no prospective or retrospective before/after study evaluating this objective is available. The impact of haemodynamic assessment to guide treatment modifications has also not been assessed, even in studies with a low level of evidence.

In view of the wide diversity of types of shock, which vary according to the toxin considered, it is legitimate to propose a clinical approach based on the standard management of shock in general, and then adapt this management to the type of toxin involved [67–70]. The example of poisonings with calcium-channel blockers and membrane-stabilizing drugs, which can indiscriminately cause vasoplegic, cardiogenic or hypovolaemic shock, can be generalized to all types of toxins [67, 71, 72]. This recommendation is also justified by the possible concomitant presence of other aetiologies for shock associated with poisoning.

Fluid resuscitation should be considered as first-line treatment for toxic shock before considering the use of catecholamines. This point was also stressed in the international guidelines on the management of calcium-channel blocker poisoning [72]. In the 2001 US guidelines [73], the experts stated that, due to the high risk of ventricular arrhythmia induced by the high doses of catecholamines sometimes required to treat certain forms of toxic shock, haemodynamic monitoring should be performed systematically with careful selection of treatments and catecholamine titration. No recommendations are available concerning the preferred monitoring technique; nevertheless, echocardiography appears to be a useful, if not essential, tool for the management of toxic shock [67, 74]. Determination of the cardiac index is the mainstay of poisoned patient monitoring in the presence of cardiovascular failure. While most patients with refractory toxic shock have low systemic vascular resistance, some patients have high vascular resistance. Central haemodynamic monitoring with measurement of systemic vascular resistance may be preferable in these patients.

### Field 4: Decontamination

#### Question 4.1: When should gastric lavage be performed in a patient with suspected pharmaceutical or recreational drug poisoning?

OPTIONAL RECOMMENDATION/GRADE 2−/STRONG CONSENSUS

**R 4.1.1: Gastric lavage should probably not be performed systematically in a patient with suspected pharmaceutical or recreational drug ingestion.**

RECOMMENDATION IN THE FORM OF AN EXPERT OPINION/STRONG CONSENSUS

**R 4.1.2: The experts suggest that gastric lavage should be performed within 1 h, in the absence of contraindications, following the ingestion of a substance not adsorbed by activated charcoal, at a presumedly toxic dose and with a high potential for organ damage.**

*Rationale* Active gastrointestinal decontamination following the ingestion of a toxin is one of the most controversial topics. Gastric lavage continues to be practiced, sometimes systematically, although its efficacy has been questioned over recent decades [75–77]. A prospective randomized controlled trial of 876 patients did not show any difference in clinical outcome according to whether or not gastric lavage was performed [76]. This lack of efficacy was recalled in the 10th consensus conference published in 1993 and then in the two position papers on gastric lavage published by the American Academy of Clinical Toxicology and the European Association of Poisons Centres and Clinical Toxicologists in 2004 and 2013 [78–80]. However, these consensus conferences concluded that gastric lavage could be beneficial in poisoned patients under certain strictly defined conditions [81]. More recent studies concluded that gastric lavage does not improve mortality after certain types of poisoning [53, 82]. Several factors determine the efficacy of gastric lavage: the nature of the toxin and its presentation (solubility, absorption rate, liquid or extended-release form), its effect on gastric emptying, the reported ingested dose and the time between ingestion and gastric lavage [23, 83, 84]. Some studies have suggested that gastric lavage may promote passage of gastric contents through the pylorus; but the results of these studies remain controversial [85]. It is generally accepted that recovery of ingested tablets remains incomplete [86]. For example, experimentally, the percentage of toxin recovered is less than 40% when gastric lavage is performed within 20 min after ingestion and falls to around 10% at the 60th min. Endoscopy performed after gastric lavage confirmed the presence of toxins in the stomach in nearly 70% of patients [87]. In a prospective study of 133 patients, gastric lavage removed an average of only 6.4% of the reported ingested dose and the authors found no correlation between the efficacy of gastric lavage and the reported ingested dose or the time since ingestion [88]. Substances not absorbed by activated charcoal that still may request gastric lavage include alcohols, ions (such as potassium and lithium) and metals (such as iron). Gastrointestinal decontamination, when indicated, must be performed very cautiously in children under the age of 6 years due to the fatal risk associated with the ingestion of certain toxins, including a unit dose of the adult dosage form [89].

Many studies have reported the high incidence of possibly severe complications of gastric lavage: oesophageal or gastric perforation, gastrointestinal bleeding, pneumoperitoneum, pneumothorax, fluid overload and hypernatraemia, hypothermia, pulmonary oedema, aspiration pneumonia, laryngospasm, tachycardia and arrythmias [53, 63, 90, 91]. All authors are also unanimous concerning the contraindications of gastric lavage, which must only be performed by teams skilled in this technique and able to act effectively in the event of complications. The main contraindications are: altered state of consciousness (without protection of the airways by intubation), ingestion of a corrosive substance or substances associated with a high risk of inhalation (hydrocarbons, foaming products), risk of gastrointestinal bleeding, unstable haemodynamics or respiratory failure.

#### Question 4.2: Should activated charcoal be administered to a patient with suspected pharmaceutical or recreational drug poisoning and, if so, as a single dose or repeatedly?

OPTIONAL RECOMMENDATION/GRADE 2−/STRONG CONSENSUS

**R 4.2.1: Activated charcoal should probably not be systematically administered to a patient with suspected pharmaceutical or recreational drug poisoning.**

RECOMMENDATION IN THE FORM OF AN EXPERT OPINION/STRONG CONSENSUS

**R 4.2.2: The experts suggest that a single dose of activated charcoal should be given in the absence of contraindication and within 1 h following the ingestion of a presumedly toxic dose of a substance adsorbed by charcoal.**

RECOMMENDATION IN THE FORM OF AN EXPERT OPINION/STRONG CONSENSUS

**R 4.2.3: The experts suggest that repeated doses of activated charcoal should be dedicated to cases of ingestion of an extended-release drug or substance with an intense enterohepatic cycle in the case of a presumedly toxic dose or potentially severe poisoning.**

*Rationale* Activated charcoal is a highly porous form of carbon with a surface area of 950 to 2000 m^2^/g capable of adsorbing substances with a molecular weight between 100 and 1000 Da. Studies in healthy subjects have shown that activated charcoal is able to adsorb certain toxins present in the gastrointestinal tract, thereby limiting their absorption and bioavailability [92, 93] or increasing their elimination. In most cases of poisoning, the ingested doses are low, the clinical effects are limited and the risk of death is also low [94]. Administration of activated charcoal should therefore be considered when there is a proven toxic risk and when a significant toxin amount is still present in the gut [95, 96].

A single dose of activated charcoal can limit the absorption of a toxin adsorbed by charcoal, provided it is administered as soon as possible after ingestion, ideally within 1 h, as the efficacy of activated charcoal declines with time [92, 95]. However, this 1-h limit appears to be highly restrictive because, on the one hand, patients are rarely admitted so soon after ingestion and, on the other hand, certain studies have shown a benefit of activated charcoal up to 4 h after ingestion of large quantities of toxins in terms of reduction of both serum levels and toxic effects [97, 98]. Administration of activated charcoal can therefore be considered beyond this 1-h period, on a case-by-case basis [92, 95].

Randomized trials of the efficacy of activated charcoal compared to symptomatic management alone or to another gastrointestinal decontamination technique present low levels of evidence [96, 99]. Administration of activated charcoal should therefore be reserved for potentially serious poisoning in addition to supportive care, although clinical studies have not demonstrated any benefit in terms of length of stay and mortality [94, 100, 101].

As adsorption of the toxin by activated charcoal is a saturable process, the commonly recommended dose is that of a 10:1 ratio between the amount of charcoal administered and the amount of toxin ingested, i.e. a dose of 25 to 100 g in adults and 1 g/kg in children [92, 95].

Repeated administration of activated charcoal can prevent absorption of toxins when this process is delayed [102] and can increase elimination by absorbing toxins diffusing from the bloodstream into the intestinal lumen and by interrupting the enterohepatic or enteroenteric cycle [95, 97, 98]. The value of this technique has been demonstrated in terms of reducing the elimination half-life for a limited number of molecules, including carbamazepine, theophylline, dapsone, quinine, and phenobarbital [102–104]. Repeated administration of activated charcoal can be considered in combination with RRT in certain indications [103]. Administration of multiple doses, compared to a single dose, may have an impact on the length of stay [104]. The recommended dose, after the first administration, is 12.5 g/h (equivalent to 50 g/4 h and 10 to 25 g/4 h in children).

Activated charcoal is contraindicated when the airways are not protected, following recent surgery, gastrointestinal perforation and ileus. Activated charcoal is readily available, inexpensive and associated with few iatrogenic effects and rare complications [105]. Randomized trials have not demonstrated any significant differences between single and multiple doses in terms of vomiting or aspiration pneumonia [100, 101]. However, patient compliance is poorer in the case of multiple doses [106].

#### Question 4.3: Should whole bowel irrigation be performed in a patient with suspected pharmaceutical or recreational drug poisoning and, if so, when and how?

RECOMMENDATION IN THE FORM OF AN EXPERT OPINION/STRONG CONSENSUS

**R 4.3: The experts suggest that the indication for whole bowel irrigation should be considered following potentially life-threatening (notably organ damage) ingestion of a substance not adsorbed by activated charcoal or an extended-release drug or in the case of body packing, taking into account the benefit-risk ratio. An expert centre or PCC opinion should be obtained.**

*Rationale* Whole bowel irrigation, using large doses of polyethylene glycol to empty the gastrointestinal tract of its contents, is considered to be an alternative method of gastrointestinal decontamination, which can be performed following ingestion of extended-release drugs, substances not adsorbed onto activated charcoal, or body packing [107]. Some studies in animals [108] or healthy subjects [109, 110] suggest that whole bowel irrigation enhances elimination of toxins (with decreased plasma concentrations and increased total body clearance).

Based exclusively on non-controlled retrospective studies, there is currently no evidence to demonstrate the efficacy of gastrointestinal decontamination by whole bowel irrigation on the clinical outcome of poisoning. The available studies concern cases of body packing of illicit drugs (cocaine, heroin, cannabis [111, 112]), and cases of poisoning by extended-release drugs [113] or substances not adsorbed onto activated charcoal (iron, lithium) [107, 114]. Several studies have also reported potentially serious complications associated with the use of this procedure (digestive disorders, anaphylaxis, respiratory distress, death) [115–119]. The possible indication for whole bowel irrigation in a case of poisoning must therefore be carefully assessed in terms of the benefit/risk balance.

### Field 5: Enhanced elimination

#### Question 5.1: When should haemodialysis be performed in order to enhance elimination of the toxin in a patient with suspected medication or recreational drug poisoning?

RECOMMENDATION IN THE FORM OF AN EXPERT OPINION/STRONG CONSENSUS

**R 5.1: The experts suggest the use of renal replacement therapy to enhance elimination of the toxin and/or prevent complications in cases of severe lithium, metformin, salicylate, phenobarbital, valproic acid or theophylline poisoning. The intermittent haemodialysis technique should be preferred. An expert centre or PCC opinion should be obtained.**

*Rationale* The clinical features of voluntary or accidental pharmaceutical or recreational drug poisoning are the result of a large number of factors, including the intrinsic properties of the toxin, the dose, the formulation, the mode of exposure, co-ingestion of other toxins, but also the patient's prior health status [120]. Despite this wide range of factors, the mortality of poisoned patients admitted to the ER or ICU is now low as a result of the efficacy of life support treatments that play a much larger role than antidotes or enhanced elimination techniques (including haemodialysis). The indication for haemodialysis in a case of poisoning must therefore take into account several different arguments [21, 22, 103, 121–123]. The risk of exposure in terms of morbidity and mortality must first be assessed, based on the properties of the toxin and the extent of exposure, by estimating the maximum ingested dose and, in some cases, plasma concentrations (see R 2.3). Prevention or reversibility of toxicity using an antidote should then be considered. The indication for RRT should be considered in a patient already presenting or at risk of developing severe clinical features, despite well-conducted supportive or specific treatments, in the absence of other alternative therapies. The decision to implement haemodialysis should be primarily guided by certain physicochemical characteristics of the toxin that may determine the capacity of haemodialysis to modify the toxicokinetics, including molecular weight (ideally less than 500 Da), volume of distribution (ideally less than 1 L/kg), plasma protein binding (ideally less than 60%) and endogenous clearance (ideally less than 4 mL/min/kg). However, even when haemodialysis can significantly alter the toxicokinetics of the toxin, the expected toxicodynamic consequences (decreased mortality, morbidity or number and severity of complications) cannot be directly extrapolated. For example, the number of candidate molecules for clearance by haemodialysis remains limited. The scientific literature is almost exclusively limited to isolated case reports or small series, which limits the level of evidence. Kinetic studies, often incomplete, do not definitively resolve the question of the net gain in terms of clearance of the various toxins. When taking the physicochemical and pharmacokinetic properties of the toxin into account, it appears reasonable to consider RRT for severe cases of lithium, metformin, salicylate, phenobarbital, valproic acid and theophylline poisoning [21–23, 48, 103, 120, 123, 124]. The indication for RRT must take the patient's creatinine clearance into account. For all other toxins, RRT use must be decided case-by-case, taking the above factors into account.

#### Question 5.2: When should an extracorporeal treatment other than haemodialysis be performed to enhance elimination of the toxin in a patient with suspected pharmaceutical or recreational drug poisoning?

RECOMMENDATION IN THE FORM OF AN EXPERT OPINION/STRONG CONSENSUS

**R 5.2: The experts suggest that an extracorporeal treatment other than intermittent (or continuous) haemodialysis should not be used to enhance clearance of the toxin in patients with pharmaceutical or recreational drug poisoning.**

*Rationale* Theoretically, compared to haemodialysis and considering substances with low volume of distribution, (1) haemofiltration is able to eliminate substances with higher molecular weights (up to 25 kDa versus 15 kDa for haemodialysis); (2) haemoperfusion, albumin dialysis and plasma exchange are able to eliminate substances strongly bound to albumin (80%) and/or high molecular weight substances, in the case of haemoperfusion (25 to 50 kDa) and plasma exchange (50 kDa); (3) exchange transfusion can eliminate substances strongly bound to red blood cells [120, 125]. However, at the present time, there is no scientific evidence to support the superior efficacy of these techniques in terms of elimination of the toxin or decreased severity of the clinical features or morbidity and mortality. Published reports of the use of these extracorporeal treatments are limited to case reports or case series [125–129]. None of these methods have been prospectively compared (except in a poor quality haemoperfusion study) with haemodialysis or the absence of extracorporeal treatment. In addition, these techniques are associated with technical difficulties, high cost, limited availability and sometimes significant iatrogenic effects [125, 130]. All of these findings suggest that, except in the context of a study protocol, an extracorporeal treatment other than haemodialysis should not be used to enhance elimination of toxins in patients with medication or recreational drug poisoning. However, in the case of dialysable toxins, it should be noted that: (1) when intermittent haemodialysis is not available and/or in the presence of extremely unstable haemodynamics, continuous renal replacement therapy is an acceptable alternative to intermittent haemodialysis [21], and (2) continuous renal replacement therapy after intermittent haemodialysis would limit "a rebound effect" for certain hydrophilic dialysable toxins for which the rate of plasma redistribution is lower than the rate of elimination of the toxin by intermittent haemodialysis (lithium, dabigatran) [120, 131, 132].

When digoxin antibody Fab fragments are administered to a patient, the experts suggest that RRT, regardless of the modality, does not need to be performed for toxicological purposes, as there is no evidence of the efficacy of RRT in these patients, in whom the elimination half-life of the Fab–digoxin complex is increased, and no evidence of a dissociation of the Fab–digoxin complex resulting in a rebound of free digoxin [122]. In patients with iron poisoning treated by deferroxamine, the experts suggest that toxicological RRT should not be performed systematically in the presence of anuric kidney failure, as, although deferoxamine and ferrioxamine (chelated iron) are dialysable, no intrinsic toxicity of ferrioxamine has been reported in the literature and there is no evidence of increased toxicity of deferoxamine in the presence of kidney failure.

#### Question 5.3: When should alkaline diuresis be performed to enhance elimination in a patient with suspected medication or recreational drug poisoning?

RECOMMENDATION IN THE FORM OF AN EXPERT OPINION/STRONG CONSENSUS

**R 5.3: The experts suggest that alkaline diuresis should be used to enhance elimination of salicylates in patients with symptomatic poisoning.**

*Rationale* Urine alkalinization, mainly using sodium bicarbonate infusion, consists of inducing a urine pH higher than 7.5, which promotes the ionized form of weak acids. Cell membranes are more permeable to non-ionized compounds than to ionized compounds. Diffusion from renal tubules to the blood is therefore decreased for ionized forms of a xenobiotic. Urine alkalinization therefore theoretically enhances the elimination of weak acids.

Salicylate, filtered by the kidney, is reabsorbed by the distal tubule, but the quantity reabsorbed decreases with increasing urine pH [133]. Numerous studies have investigated the effect of urine alkalinization in acetylsalicylic acid poisoning. However, these studies have a very limited clinical relevance due to major methodological biases (in particular, observational descriptive series, vague characterization of study populations, implementation of associated treatments, inaccurate or nonspecific quantification of salicylate concentrations, no assessment of clinical consequences). However, three studies should be mentioned. In a crossover study in six healthy subjects after oral intake of 1.5 g of sodium salicylate, Vree et al. [134] compared the respective effects of urine acidification and urine alkalinization [135]. Alkalinization significantly decreased the elimination half-life and increased the total clearance of salicylate. In another study comparing sixteen moderately severely poisoned patients treated with oral hydration (urine pH, 6.1–0.4) with six patients treated with hydration and intravenous alkalinization (urine pH, 8.1 ± 0.5), Prescott et al. [136] showed that this second type of treatment increased the renal clearance of salicylates and significantly decreased their elimination half-life. This beneficial effect of increasing urine pH was also reported by Morgan et al. [137] when indirectly comparing two sets of patients with moderate and extreme urinary pH of 6.60 [6.00–7.05] and 7.88 [7.60–8.00], respectively. Several case reports have also demonstrated the value of urine alkalinization [138–140]. Thus, despite their poor quality, several studies have confirmed the enhanced urinary elimination of salicylates induced by urine alkalization. These results have been repeatedly considered to be sufficient to recommend urine alkalization as an adequate first-line treatment for salicylate poisoning, avoiding the need for haemodialysis [133, 140–142].

### Field 6: Place of antidotes

#### Question 6.1: Should a comatose patient and/or a patient in respiratory failure with suspected complicated benzodiazepine and/or opioid poisoning by treated by an antidote or intubated and mechanically ventilated?

OPTIONAL RECOMMENDATION/GRADE 2+/STRONG CONSENSUS

**R 6.1.1: Flumazenil should probably be used in a comatose patient with suspected benzodiazepine overdose to avoid intubation/mechanical ventilation which would otherwise be justified by the patient’s conditions. The use of flumazenil is contraindicated in cases of co-poisoning with a proconvulsive drug or in patients with a known history of epilepsy.**

STRONG RECOMMENDATION/GRADE 1+/STRONG CONSENSUS

**R 6.1.2: Naloxone should be used in a comatose patient with suspected opioid overdose to avoid intubation/mechanical ventilation which would otherwise be justified by the patient’s condition.**

*Rationale* The use of flumazenil in a comatose patient with suspected benzodiazepine overdose essentially plays a diagnostic role, limiting the use of invasive diagnostic or therapeutic procedures [143]. The use of flumazenil can limit the need for intubation by improving the level of consciousness of patients with true benzodiazepine poisoning [144, 145]. The major side effects of flumazenil, namely ventricular arrhythmias or tonic–clonic seizures, are rare and mainly observed in the context of co-poisoning with tricyclic antidepressants or in patients with chronic high-dose benzodiazepine use [146, 147]. The use of flumazenil therefore requires continuous monitoring of the patient. No published study has compared the use of flumazenil and endotracheal intubation in terms of the incidence of aspiration pneumonia. When intubation remains indicated despite administration of flumazenil, it should be performed without delay. The titration dosage of 0.1 mg flumazenil every 30 s until the patient wakes up, followed by continuous intravenous infusion with a syringe driver at an hourly dosage equal to the titration dose with monitoring in CCU appears to be a safe approach [146, 147]. The titration dose should achieve a level of consciousness compatible with effective ventilation and airway protection.

The mortality of opioid overdose is increasing in the US [148] and, to a lesser extent, in Europe [149], and concerns a young population. No randomized trials have compared the use of naloxone *versus* endotracheal intubation in opioid-related coma. However, many good quality cohorts have reported zero mortality after the use of naloxone in cases of opioid overdose [150–152]. Naloxone allows awakening of the poisoned patient, restoration of a respiratory rate > 15/min (in adults) and return home just a few hours after management initiation [151, 152]. The titration dosage is 0.04 mg (0.01 mg/kg in children) every 60 s until the patient wakes up. Its short duration of action (20 to 30 min) is usually only able to reverse the peak effect of heroin and immediate-release morphine [148]. Continuous intravenous infusion with a syringe driver at an hourly dose equal to half the titration dose should therefore be proposed in the case of poisoning by another opioid (including methadone) or an extended-release opioid [148] and admission to the CCU is then indicated. The efficacy of naloxone remains controversial in buprenorphine poisoning [153, 154] and should be used with caution in tramadol poisoning, due to the unresolved question of whether or not naloxone increases the seizure risk. Adverse effects of naloxone are very rare with an uncertain causality, apart from the risk of withdrawal syndrome after a non-titrated injection [148, 155].

#### Question 6.2: When should *N*-acetylcysteine be administered to a patient with suspected acetaminophen poisoning? Should treatment be guided by the nomogram?

STRONG RECOMMENDATION/GRADE 1+/STRONG CONSENSUS

**R 6.2.1: N-acetylcysteine should be administered after a single ingestion of acetaminophen at a known time when serum acetaminophen concentrations after the 4th hour post-ingestion are situated above the line of liver toxicity according to the Rumack and Matthew nomogram (150 mg/L at the 4th hour).**

RECOMMENDATION IN THE FORM OF AN EXPERT OPINION/STRONG CONSENSUS

**R 6.2.2: The experts suggest that N-acetylcysteine should be routinely administered in the presence of a high suspicion of toxic acetaminophen dose ingestion without interpreting serum acetaminophen concentrations according to the Rumack and Matthew nomogram in the following cases:**

Unknown time of ingestion. Treatment is continued as long as serum acetaminophen is not equal to zero or if ALAT is elevated.

Documented risk factors (chronic liver disease, nutritional deficiency). Treatment is continued if serum acetaminophen is not below the lower detection level or if ALAT is elevated.

Delayed admission, after the 24th hour post-ingestion with elevated ALAT.

Repeated ingestion of supratherapeutic doses of acetaminophen. Complete treatment should be administered and continued in the presence of elevated ALAT.

*Rationale* *N*-Acetylcysteine has been the recognized antidote for acetaminophen poisoning since 1979 [49, 156]. Only one randomized study, published in 1991, compared *N*-acetylcysteine *versus* placebo in subjects with liver failure after acetaminophen poisoning and showed a better survival rate with *N*-acetylcysteine [157]. A large number of case series and observational studies have subsequently confirmed the efficacy of N-acetylcysteine [158, 159]. For ethical reasons, no randomized trial can be conducted to determine the real value of administering *N*-acetylcysteine in acetaminophen poisoning.

Although never supported by randomized trials, the Rumack and Matthew nomogram is extensively used worldwide to determine the indication for *N*-acetylcysteine following the ingestion of a single toxic acetaminophen dose [158]. The treatment threshold varies from country to country [160, 161]. In France and almost all other Western countries (with the exception of United Kingdom and Denmark), the treatment threshold is 150 mg/L at the fourth hour post-ingestion [162]. Recent retrospective data suggest that potentially fatal poisonings can occur at thresholds below 150 mg/L [163, 164]. Pending further studies, particularly studies on new biomarkers, the treatment threshold should not be changed.

In addition to a single ingestion of a toxic dose of acetaminophen, other situations are associated with a particularly high risk. Two observational studies suggest that repeated ingestion of toxic doses of acetaminophen or cases of delayed presentation after ingestion of a single dose are associated with poor prognosis [165, 166]. Subjects with underlying liver disease (including steatosis), chronic alcoholism or treatment with cytochrome P450 enzyme inducers are at higher risk of toxicity [167, 168]. In these high-risk settings, the decision to administer *N*-acetylcysteine preventively must be broader, without necessarily relying on the Rumack and Matthew nomogram.

Current research focuses on simplified *N*-acetylcysteine dosing regimens [169, 170]. A randomized study showed that a simplified protocol reduced the frequency of side effects of *N*-acetylcysteine [171], although was unable to determine its efficacy due to lack of power. Two recent observational studies have reported similar results [172, 173]. In the current state of knowledge and pending non-inferiority studies, the so-called Prescott regimen should be preferred (150 mg/kg in 1 h—loading dose—followed by 50 mg/kg over four hours and then 100 mg/kg over 16 h, by intravenous infusion) [49]. Already used in the UK and Australia, a simplified protocol will very probably be recommended in the near future. In massive poisoning, doubling *N*-acetylcysteine dose of the third bag has been advised.

#### Question 6.3: When should an antidote (when one exists) be administered to a patient with medication or recreational drug poisoning?

RECOMMENDATION IN THE FORM OF AN EXPERT OPINION/STRONG CONSENSUS

**R 6.3.1: The experts suggest that, when an antidote exists, it should not be administered systematically.**

OPTIONAL RECOMMENDATION/GRADE 2+/STRONG CONSENSUS

**R 6.3.2: Following exposure to a functional toxin, an antidote should probably be administered in the presence of signs of severity.**

OPTIONAL RECOMMENDATION/GRADE 2+/STRONG CONSENSUS

**R 6.3.3: Following exposure to a toxic dose of a toxin causing organ damage, an antidote should probably be administered according to its specific modalities (Table ****4****), preferably before the onset of organ damage.**

**Table 4:** Indications and recommended availability times for the main antidotes

Antidote	Toxin	Indications	Availability	Evidence level
Folinic acid (l-folinic acid)	Methotrexate	SID–1000 mg/m^2^ (taking serum methotrexate levels into account) Kidney failure	2 h	Expert opinion
Digoxin antibodies [248]	Digoxin	*Semimolar neutralization:* bradycardia ≤ 50 bpm refractory to 1 mg of atropine i.v.; atrioventricular block (regardless of degree); serum potassium ≥ 4.5 mmol/L *Molar neutralization*: ventricular arrhythmias (ventricular fibrillation or ventricular tachycardia); severe bradycardia ≤ 40 bpm refractory to 1 mg of atropine i.v.; serum potassium ≥ 5.5 mmol/L; mesenteric infarction; cardiogenic shock	Expert centre	Expert opinion
Methylene blue (methylthioninium chloride, Proveblue^®^)	Sulphones Sulphonamides Lidocaine, prilocaine Poppers	Methaemoglobinaemia ≥ 20% or signs of tissue hypoxia	Immediate	Expert opinion
Carbopeptidase G2 (Voraxase^®^)	Methotrexate	Serum methotrexate ≥ 1 µmol/L at H48 with kidney failure	Expert centre	Expert opinion
Deferoxamine Desferal^®^	Iron	SID ≥ 150 mg/kg and/or Signs of poisoning Serum iron at H2–H4 ≥ 500 µg/dL Metabolic acidosis	> 2 h	Expert opinion
Diazepam [249]	Chloroquine	In the presence of a single risk factor for poor prognosis: SID ≥ 4 g or systolic blood pressure ≤ 100 mmHg, or QRS ≥ 100 ms In combination with intubation and adrenaline	Immediate	Expert opinion
Flumazenil [250]	Benzodiazepines	Coma and/or acute respiratory failure requiring intubation	Immediate	Grade 2
Idarucizumab Praxbind^®^	Dabigatran	Severe haemorrhage Surgical emergency	< 2 h	Grade 2
l-Carnitine	Valproic acid	Severe poisoning with hyperammonaemia or hyperlactataemia Plasma concentration ≥ 850 mg/L	< 2 h	Expert opinion
*N*-Acetylcysteine	Acetaminophen	Serum acetaminophen greater than 150 mg/L at H4 (Rumack and Matthew's nomogram) Unknown time of intake or impaired level of consciousness (continue if serum acetaminophen remains detectable or elevation of ALAT) Susceptibility factor (chronic liver disease, nutritional deficiency; continue if serum acetaminophen remains detectable or elevation of ALAT) Delayed admission more than H24 post-exposure with elevated ALAT Repeated use of supratherapeutic doses of acetaminophen (continue if elevation of ALAT)	2 h	Grade 1+ Expert opinion
Naloxone [251]	Opioids	Coma and/or respiratory depression requiring intubation	Immediate	Grade 1+
Neostigmine	Non-depolarizing muscle relaxants Anticholinergics	Respiratory distress (TOF > 0/4) Severe anticholinergic syndrome	Immediate	Expert opinion
Octreotide [252]	Sulphonylureas	Symptomatic hypoglycaemia	Immediate	Grade 2
PPSB	Vitamin K antagonist anticoagulants Direct oral anticoagulant	Severe or potentially severe haemorrhage Urgent surgery In combination with vitamin K	Immediate	Expert opinion
Sugammadex	Rocuronium Vecuronium	Respiratory distress	Operating room	Grade 2
Protamine sulphate	Unfractioned heparin and low-molecular weight heparins (less effective)	Severe haemorrhage Surgical emergency	< 2 h	Expert opinion
Vitamin B6	Isoniazid	SID > 2 g with seizures	< 2 h	Expert opinion
Vitamin K1	Vitamin K antagonist anticoagulants	INR ≥ 6 And/or severe haemorrhage And/or urgent surgery In combination with PCC	Immediate	Expert opinion

*ALAT* alanine aminotransferase, *SID* supposed ingested dose, *INR* international normalized ratio, *i.v*. intravenous, *PCC* prothrombin complex concentrate, *TOF* train-of-four (stimulations)

*Rationale* Antidotes are drugs that are able to alter the kinetics and/or effects of the toxin. Administration of an antidote provides a clinical benefit for the poisoning patient [174]. The indication for an antidote must be guided by the duration of action of the toxin and the antidote, the expected benefit and the iatrogenic risk of the antidote [175]. Antidotes have different mechanisms of action, modifying either the toxicokinetics or the toxicodynamics, or both mechanisms may sometimes be involved. These variable mechanisms of action explain why the expected objectives of administration of an antidote may vary and that the useful period of administration of an antidote may depend on its mechanism of action. For toxins that cause organ damage (e.g. acetaminophen), the antidote should be used prior to onset of organ damage, otherwise the efficacy and clinical value of the antidote may be decreased or even eliminated [176].

PCC support is useful when determining the indication for and the modalities of administration of an antidote, its availability, its modalities of use, the possibility of re-administration, and to ensure monitoring of its efficacy and side effects [177–179]. It is highly recommended for health care professionals to report poisoning cases that require administration of a rare and/or expensive antidote to a PCC [180]. Studies with a high level of evidence concerning the use of antidotes are rare. However, due to the life-threatening risk associated with certain forms of poisoning, the use of antidotes appears to be justified. Table 4 summarizes the main antidotes available in France and their respective indications. This table is not exhaustive. Other antidotes not listed in this table may be considered after consultation with a PCC in a case of serious poisoning and/or a little-known toxin.

### Field 7: Specificities related to recreational drug poisoning

#### Question 7.1: What are the clinical and laboratory (other than toxicological) signs of severity in a patient with suspected recreational drug poisoning?

RECOMMENDATION IN THE FORM OF AN EXPERT OPINION/STRONG CONSENSUS

**R 7.1: The experts suggest that a patient with suspected recreational drug poisoning should be examined for the presence of clinical signs of severity and that complementary tests guided by the type or types of drugs used should be performed (Table ****5****).**

**Table 5:** Signs of severity of recreational drug poisoning

Drug	Clinical severity sign	Paraclinical examinations in search of gravity	
Amphetamines [253–255]	Hyperthermia Neurological signs suggestive of stroke Chest pain suggestive of myocardial infarction Seizures	Serum electrolytes; urea/creatinine; CPK; troponin Arterial blood gases Electrocardiogram Signs to look for: Metabolic acidosis Hyponatraemia Rhabdomyolysis Kidney failure Ischaemia/myocardial necrosis Arrythmia and conduction disorder	Expert opinion
Benzodiazepines	Respiratory depression	Arterial blood gases	Expert opinion
Cannabis [256, 257]	Angina	CPK; troponin Electrocardiogram Search for myocardial ischaemia/necrosis	Expert opinion
Synthetic cannabinoids [258, 259]	Encephalopathy and extreme agitation leading to endangerment of the patient Seizures Angina	Serum electrolytes; urea-creatinine CPK; troponin Electrocardiogram Look for signs of: Kidney failure Electrolyte disorders Myocardial ischaemia/infarction	Expert opinion
Synthetic cathinones [258, 260, 261]	Life-threatening encephalopathy and extreme agitation Seizures Cardiorespiratory collapse Respiratory depression Hyperthermia	Serum electrolytes; urea, creatinine CPK; troponin Arterial blood gases Electrocardiogram Look for signs of: Hyponatremia Abnormal serum potassium Kidney failure Rhabdomyolysis Myocardial ischaemia/infarction	Expert opinion
*Magic mushrooms* [262–264]	Life-threatening encephalopathy and hallucinations	Electrocardiogram (adrenergic signs)	Expert opinion
Cocaine [265, 266]	Cardiorespiratory collapse Angina Respiratory failure and aspiration	Electrocardiogram CPK; troponin Look for signs of: Myocardial ischaemia/infarction Arrythmia and conduction disorders (membrane-stabilizing effect)	Expert opinion
Codeine [267, 268]	Respiratory depression Associated toxicity of acetaminophen (when taken concomitantly)	Serum electrolytes; urea, creatinine Transaminases, bilirubin, PT Look for signs of: Hepatocellular insufficiency Kidney failure	Expert opinion
Crack [269]	Cardiorespiratory collapse Angina Respiratory failure and aspiration (crack lung)	Electrocardiogram CPK; troponin Look for signs of: Myocardial ischaemia/infarction Arrythmia and conduction disorders (membrane-stabilizing effect)	Expert opinion
Lysergic acid diethylamide (LSD) [270, 271]	Cardiorespiratory collapse Respiratory depression	Serum electrolytes; urea, creatinine; CPK PT Look for signs of: Kidney failure Rhabdomyolysis Clotting disorders	Expert opinion
GHB/GBL [272]	Respiratory depression Seizures	CPK Electrocardiogram (sinus bradycardia, atrioventricular block, U waves)	Expert opinion
Heroin [273]	Respiratory depression	Serum electrolytes; urea, creatinine; CPK Electrocardiogram Look for signs of: Rhabdomyolysis Kidney failure Myocardial ischaemia/infarction	Expert opinion
Ketamine/phencyclidine [274]	Respiratory depression	Serum electrolytes; urea, creatinine; CPK Transaminases, bilirubin; PT Look for signs of: Rhabdomyolysis Kidney failure Hyponatraemia Hepatocellular insufficiency	Expert opinion
Poppers [275]	Cardiorespiratory collapse	Methaemoglobinaemia Arterial blood gases; serum lactate	Expert opinion
Tramadol [276, 277]	Seizures Respiratory failure Cardiocirculatory collapse Associated toxicity of acetaminophen (when taken concomitantly)	Electrocardiogram (membrane-stabilizing effect) Serum electrolytes; urea, creatinine Transaminases, bilirubin, PT Look for signs of: Hepatocellular insufficiency Kidney failure	Expert opinion

*CPK* creatine phosphokinase, *GHB* γ-hydroxybutyric acid, *GBL* γ-butyrolactone, *PT* prothrombin time

*Rationale* The severity of recreational drug poisoning may be related to the effects of the toxin or nonspecific poisoning complications. The initial assessment of the prognosis of recreational drug poisoning must take into account the characteristics of the toxins consumed, the reported dose used, the formulation, the patient's comorbidities, the time at which the patient was managed and the presence of any complications. The clinician must take into account drug combinations used due to additive or synergistic effects. The patient's initial asymptomatic presentation does not necessarily indicate a favourable prognosis. There is no direct relationship between the presumed drug-related coma depth and the final poisoning prognosis in the ICU. Due to the difficulties of identifying the toxins involved and their uncertain causality when multiple substances are involved, few studies have assessed predictive factors of the morbidity and mortality of recreational drug poisoning. Table 5 summarizes the symptoms and clinical and laboratory signs of severity for each class of drug that must be detected as early as possible.

#### Question 7.2: In a patient with recreational drug poisoning, can systematic analytical identification optimize management, improve prognosis and/or allow implementation of preventive public health actions?

RECOMMENDATION IN THE FORM OF AN EXPERT OPINION/STRONG CONSENSUS

**R 7.2.1: The experts suggest that systematic analytical identification (especially NPS) does not improve patient management.**

RECOMMENDATION IN THE FORM OF AN EXPERT OPINION/STRONG CONSENSUS

**R 7.2.2: The experts suggest that systematic analytical identification (particularly NPS) should be performed in the context of an alert network.**

**Fig. 1:**
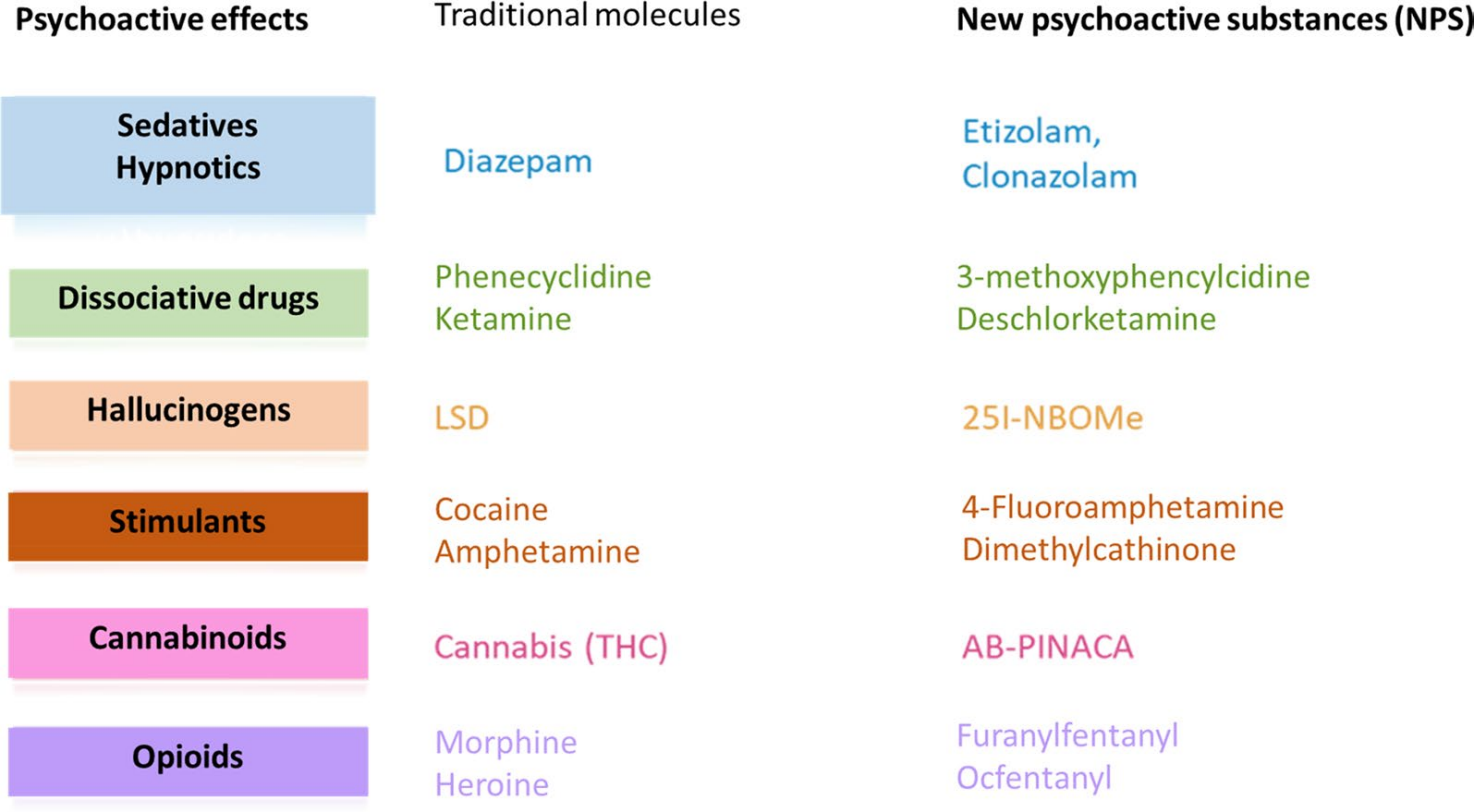
Examples of new psychoactive substances (NPS) mimicking the psychoactive effects of "traditional" molecules

**Fig. 2:**
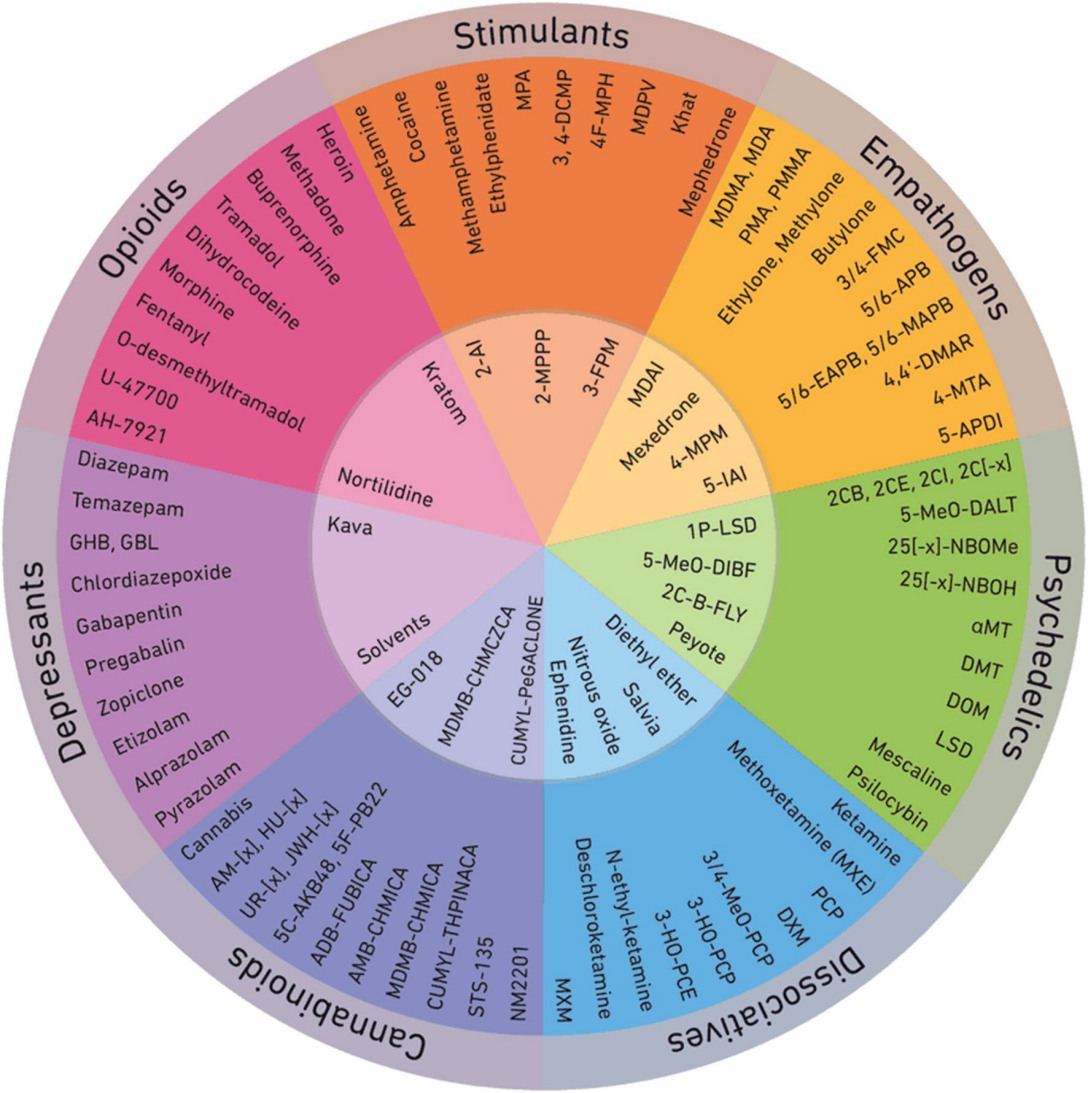
The Drugs Wheel, a new model for substance awareness (UK version 2.0.7 dated 08/09/2018—www.thedrugswheel.com)

*Rationale* Although no published studies have addressed this issue, the available data suggest that analytical identification of recreational drugs is of limited value for patient management and generally cannot be performed routinely due to the mismatch between the emergency setting and the time required to obtain the results [181–185]. However, retrospective identification is useful to more clearly understand the observed symptoms and signs, and, as a result of the acquired knowledge, contributes to the improvement of care [183, 186], medical treatment (particularly, in addiction medicine [187, 188]) and implementation of preventive actions [189, 190]. Epidemiologically, analytical identification contributes to the limited and not always up-to-date data [191], and provides support for an early warning system for emerging substances [183, 192, 192]. The classification and the main effects of NPS are described in the inset and in Figs. 1 and 2. The new psychoactive substances, commonly known as NPS, have been defined by the United Nations Office on Drugs and Crime (UNODC) as “substances of abuse, either in a pure form or a preparation, that are not controlled by the 1961 Single Convention on Narcotic Drugs or the 1971 Convention on Psychotropic Substances, but which may pose a public health threat”. In 2018, a total of about 650 molecules had been identified in Europe and just over 300 molecules had been identified in France, belonging to 11 different chemical families. Several classifications have been proposed. One classification based on chemical structure identifies the following families of substances:

- Aminoindanes
- Arylalkylamines
- Benzodiazepines
- Synthetic cannabinoids
- Cathinones
- Indolalkylamines
- Synthetic opioids
- Phenethylamines
- Piperazines
- Piperidines and pyrrolidines

The chemical structures of these substances may be similar to that of traditional substances, but are sometimes different. NPS are designed to mimic the effects of already known medicinal products or drugs (Fig. 1) and to circumvent legislation. A classification of NPS based on the desired psychoactive effects compared to traditional psychoactive substances is also proposed (Fig. 2).

Many cases of NPS poisoning, involving various substances, have been reported in France over recent years, but only limited published data are available. For example, in the DRAMES (Décès en relation avec l’abus de médicaments et de substances [deaths related to drug and substance abuse]) survey set up by the ANSM (Agence nationale de sécurité du médicament et des produits de santé [French Agency for the Safety of Health Products])—annual prospective study), 36 deaths involving NPS have been reported since 2012. Cathinones (3-MMC, 4-MEC, butylone, MDPV, mephedrone, methylone, mexedrone, penterone, a-PVP) were the substances most often implicated in these deaths in each year of the survey. Other families are also involved: benzofurans (5-APB, 5-APDB, 5-MAPB), arylcyclohexylamines (MXE, MXP), designer benzodiazepines (diclazepam, deschloroetizolam), NBOMe (25C-NBOMe), synthetic opioids (ocfentanil), piperazines (ethylphenidate), other substances (3FPM, MPA).

#### Question 7.3: Does the use of specific treatments alter the patient's prognosis after NPS poisoning?

RECOMMENDATION IN THE FORM OF AN EXPERT OPINION/STRONG CONSENSUS

**R 7.3: The experts suggest that cyproheptadine should be administered for toxic hyperthermia, in combination with symptomatic treatment, in a patient with NPS (especially cathinone) poisoning.**

*Rationale* Following the use of psychostimulant or hallucinogenic NPS, the toxic features comprise adrenergic signs (tachycardia, hypertension, restlessness, mydriasis), encephalopathy (confusion, hallucinations), serotonergic signs (myoclonus, fever) and/or organ failure [193–196]. There is a high risk of neurological complications (coma, seizures, stroke), as well as a risk of cardiovascular, respiratory, renal (tubular necrosis due to rhabdomyolysis, tubulo-interstitial nephritis with halogenated cannabinoids), liver and/or haematological failure (disseminated intravascular coagulation, haemorrhage due to contamination of synthetic cannabinoids with vitamin K antagonist rat poisons) [197, 198]. Opioid syndrome is observed after consumption of a central nervous system depressant NPS, although atypical features have been reported (tachycardia, hypertension, kidney failure) [199]. The duration of clinical manifestations depends on the elimination half-life of the substance, which is often prolonged at high doses and in the presence of kidney or liver failure. However, it is not easy to identify the toxin responsible based solely on toxidromes, which highlights the role of specialized toxicology analysis.

Cases of NPS poisoning are generally managed in the emergency room and more rarely in the ICU. Management consists of supportive care combining rehydration, sedation of agitated patients by benzodiazepines or neuroleptics, anticonvulsants in the presence of seizures, antiemetics in cannabinoid hyperemesis syndrome, endotracheal intubation in patients with disorders of consciousness or organ failure, mask oxygenation or mechanical ventilation in the presence of respiratory failure, fluid resuscitation and catecholamines in the presence of circulatory failure. RRT may be useful to treat life-threatening fluid and electrolyte disorders, but does not accelerate elimination of the toxin. Malignant hyperthermia and severe serotonergic toxicity may require external cooling or even muscle relaxation after sedation and mechanical ventilation. Oral or intragastric administration of cyproheptadine (5HT-2A and 5HT-2C serotonin receptor antagonist) is recommended for drug-induced hyperthermia (typical regimen: loading dose of 12 mg followed by 4–8 mg/6–8 h); but the benefit of this treatment, by analogy with its efficacy in 3,4-methylenedioxy-methamphetamine (MDMA) serotonergic syndrome), is based exclusively on case reports [200]. The place of dantrolene has not been clearly established; however, dantrolene seems inefficient to suppress the increase in body temperature and prevent the death in rodent models of serotonin syndrome [201]. Neurorespiratory depression induced by opioid NPS appears to be reversible with naloxone, although higher doses may be necessary to avoid endotracheal intubation [199]. Topical application of capsaicin has recently been reported to be useful to treat cannabinoid hyperemesis syndrome refractory to the usual antiemetics [202].

### Field 8: Specificities of cardiotoxicant poisoning

#### Question 8.1: Should an antidote be administered to a patient with presumed cardiotoxicant poisoning and, if so, which antidote should be administered?

RECOMMENDATION IN THE FORM OF AN EXPERT OPINION/STRONG CONSENSUS

**R 8.1.1: The experts suggest that an antidote should be administered to all patients with presumed cardiotoxicant poisoning with signs of clinical or prognostic severity, according to the specific modalities of each molecule (Table ****6****).**

**Table 6:** Main antidotes for cardiovascular drugs

Antidote	Toxin	Indication	Availability	Comments
Atropine	Negative chronotropic effects	Bradycardia QT prolongation	Immediate	Expert opinion
Hypertonic sodium bicarbonate	Membrane-stabilizing effects	QRS ≥ 120 ms and MAP ≤ 65 mmHg	Immediate	Expert opinion
Calcium salts	Calcium-channel blockers	HR ≤ 60 bpm MAP ≤ 65 mmHg	Immediate	Expert opinion
Catecholamine	Polyvalent	Shock	Immediate	Grade 2
Digoxin antibody Fab fragments	Digoxin		< 2 h	Grade 2
Glucagon	Beta-blockers	Bradycardia	< 2 h	Expert opinion
Isoprenaline	Beta-blockers (sotalol) Negative chronotropic effects: calcium-channel blockers	QT prolongation Torsades de pointes Bradycardia	Immediate	Expert opinion
Insulin–glucose	Calcium-channel blockers Beta-blockers	Bradycardia MAP ≤ 65 mmHg	Immediate	Expert opinion

*FC* heart rate, *MAP* mean arterial pressure, *SID* supposed ingested dose

STRONG RECOMMENDATION/GRADE 1+/STRONG CONSENSUS

**R 8.1.2: Fluid resuscitation should be performed as first-line procedure in the presence of toxin-induced hypotension.**

STRONG RECOMMENDATION/GRADE 1+/STRONG CONSENSUS

**R 8.1.3: A catecholamine should be administered if fluid resuscitation has failed in the presence of toxin-induced shock.**

RECOMMENDATION IN THE FORM OF AN EXPERT OPINION/STRONG CONSENSUS

**R 8.1.4: In patients with toxin-induced shock, in the absence of haemodynamic assessment, the experts suggest first-line treatment with norepinephrine or epinephrine depending on the clinical presentation and the toxin involved.**

*Rationale* Atropine increases heart rate via its action on muscarinic acetylcholine receptors and reactivates adenylate cyclase (pathway not involving beta-adrenergic receptors). Atropine is recommended as first-line treatment to reverse isolated toxic bradycardia due to beta-blockers or calcium-channel blockers [71, 72, 203–205]. Acceleration of the heart rate after a single dose of atropine makes the diagnosis of severe poisoning unlikely. In contrast, the absence of atropine efficacy reflects complete adrenergic blockade, indicating the need to use other antidotes. Atropine is administered as a direct IV bolus of 0.5 mg (0.02 mg/kg, maximum of 1 mg in children), repeated every 3 to 5 min without exceeding a dose of 1.5 mg, for a target heart rate of 60 bpm.

Isoprenaline is a non-selective beta-1/beta-2 adrenergic receptor agonist. There are no published randomized controlled trials of the use of isoprenaline in the prevention or treatment of toxic torsades de pointes. The risk of torsades de pointes in the presence of QT prolongation can be most reliably estimated by the Rautaharju correction of measured QT [QTcRTH = QT * (120 + heart rate)/180] or by using an adequate nomogram [206–209].

A number of sodium channel blockers exert membrane-stabilizing effects. This toxic effect is identified by widening of the QRS complex on the ECG, which constitutes an indication for hypertonic sodium bicarbonate therapy [210–212]. Experimental data suggest a cumulative efficacy of alkalinization and hypertonic saline solution [213]. However, no randomized controlled trial has confirmed the clinical efficacy and improvement of the patient's prognosis as a result of this treatment. In the presence of QRS widening (QRS ≥ 120 ms) with or without hypotension, it is proposed to initially administer a bolus of 1 to 2 mL/kg of hypertonic 8.4% sodium bicarbonate solution (not exceeding 250 mL per administration in children weighing less than 20 kg), while monitoring serum potassium.

Experimental data have shown variable chronotropic and inotropic (catecholamine-like) effects of glucagon [214, 215]. Glucagon is generally not used alone, but in combination with other catecholamines [71]. In adults, it is recommended to inject a bolus dose of 5 to 10 mg over 1 to 2 min (in children, 0.05–0.15 mg/kg–maximum 1 mg). The efficacy of glucagon on heart rate and/or blood pressure should be measured over the following minutes. When the bolus is effective, glucagon can be administered by continuous infusion at a dose of 10 mg/h (0.1 mg/kg/h in children).

High-dose insulin euglycaemic therapy is proposed for the treatment of calcium-channel blocker and beta-blocker poisonings. No randomized controlled trial has confirmed the efficacy of this treatment, but many case reports or case series have reported its efficacy on haemodynamic parameters [216–222]. Insulin is initially administered as a bolus of 1 IU/kg and then continuously at a dose of 1 IU/kg/h. Efficacy is confirmed by haemodynamic stabilization after several minutes or several hours [223]. In the absence of initial efficacy, the insulin infusion can be increased by steps up to 10 IU/kg/h [221]. Hourly blood glucose monitoring at the bedside is mandatory to avoid any risk of hypoglycaemia related to the very elevated insulin doses used, although such a risk in severely calcium-channel blocker-overdosed patients is relatively rare.

Intuitively, calcium supplementation could be considered to be an antidote for calcium-channel blocker poisoning, but the only available data are based on case reports [224]. A single observational study has reported the efficacy of a calcium supplement on mean arterial blood pressure [225]. Calcium chloride should be preferred to calcium gluconate, because the amount of calcium delivered is threefold higher for the same volume administered. Calcium is administered as a bolus of 10 mL of 10% calcium chloride solution every 2 to 3 min up to a dose of 50 mL, possibly followed by continuous infusion at a dose of 10 mL/hour. Serum ionized calcium should be monitored to avoid exceeding a concentration of 2 mmol/L.

Digoxin-specific antibody (Fab) fragments represent the treatment of choice for reversing cardiac and non-cardiac signs of severe digoxin poisoning, as this treatment has decreased the mortality from 20–30% to 5–8% [51, 226]. The quantity of Fab to be administered is based on criteria of severity or poor prognosis.

Unlike non-toxic shock, in which the use of catecholamines is now relatively well defined on the basis of numerous studies [69, 227], no randomized controlled trials have assessed the use of catecholamines in toxin-induced shock. When comparing the clinical efficacy of various catecholamines, epinephrine appears to be the most effective agent, followed by norepinephrine [228]. The initial choice can be guided by the clinical features: epinephrine should be preferred in the presence of shock with low heart rate or conduction disorders on ECG, while norepinephrine is preferable for shock with rapid heart rate [229]. Table 6 summarizes the main antidotes and their indications in cardiotoxicant poisonings (Additional file 1).

#### Question 8.2: Should intravenous lipid emulsion (ILE) be administered to a patient with cardiotoxicant poisoning and, if so, according to which modalities?

RECOMMENDATION IN THE FORM OF AN EXPERT OPINION/STRONG CONSENSUS

**R 8.2.1: The experts suggest that ILE should not be administered to patients with cardiotoxicant poisoning in the absence of signs of clinical severity or poor prognosis.**

RECOMMENDATION IN THE FORM OF AN EXPERT OPINION/STRONG CONSENSUS

**R 8.2.2: The experts suggest that ILE should be administered to patients with local anaesthetic poisoning with signs of severity in addition to resuscitation measures.**

RECOMMENDATION IN THE FORM OF AN EXPERT OPINION/STRONG CONSENSUS

**R 8.2.3 The experts suggest that ILE should not be administered in the case of poisoning with non-fat-soluble cardiotoxicants.**

OPTIONAL RECOMMENDATION/GRADE 2+/STRONG CONSENSUS

**R 8.2.4: ILE should probably be administered, after failure of standard resuscitation measures, in the case of immediately life-threatening fat-soluble cardiotoxicant poisoning prior to ECMO.**

RECOMMENDATION IN THE FORM OF AN EXPERT OPINION/STRONG CONSENSUS

**R 8.2.5: The experts suggest that ILE should not be administered to prevent possible deterioration.**

*Rationale* The literature on ILE is characterized by heterogeneous results and administration protocols (time of administration, dose regimen, duration) and the presence of methodological biases, resulting in a low level of evidence. The beneficial effects of ILE therapy in systemic local anaesthetic overdose (particularly bupivacaine), reported in experimental studies and confirmed by case reports with a favourable benefit/risk balance, justify the use of ILE in addition to standard resuscitation measures [230, 231].

Based on the lipid theory demonstrated in pharmacokinetic studies [232, 233], many case reports have reported the efficacy of ILE therapy in cases of poisoning by lipophilic molecules (octanol/water partition coefficient or logP > 2; examples: verapamil, diltiazem, propranolol, amitriptyline, bupropion, haloperidol) [234]. However, in view of the low level of evidence, the already established benefit of other therapies (high-dose insulin euglycaemic therapy, molar sodium bicarbonate, circulatory assistance) and the side effects of ILE, ILE should only be proposed after failure of other therapies [235–237].

Several types of ILE and treatment protocols are available, but no comparative data have been published [238]. No studies have defined the optimal dosage, duration of administration and order of initiation. Until good quality studies become available, ILE therapy is administered according to the protocol most commonly used in the context of local anaesthetic poisoning (Intralipid^®^ 20%, bolus of 1.5 mL/kg followed by an infusion of 0.25 mL/kg/min), after failure of conventional therapies, and continued until resolution of signs of severity or up to the generally accepted maximum dose of 10 mL/kg [237]. No studies support the use of ILE rather than other therapies. ILE administration must therefore not delay the use of more conventional therapies such as extracorporeal life support or transfer of these patients to an expert centre [239].

#### Question 8.3: Should extracorporeal life support be used in a patient with cardiotoxicant drug poisoning with cardiovascular failure or cardiac arrest and, if so, according to which modalities?

RECOMMENDATION IN THE FORM OF AN EXPERT OPINION/STRONG CONSENSUS

**R 8.3: The experts suggest that extracorporeal life support using veno-arterial (VA) ECMO should be implemented to improve survival in patients with cardiotoxicant poisoning, in refractory cardiac arrest or cardiovascular failure refractory to pharmacological treatment.**

*Rationale* No randomized prospective studies have evaluated the role of VA-ECMO in the treatment of circulatory failure in patients with cardiotoxicant poisoning. A retrospective study analysed a group of patients treated with VA-ECMO versus a control group. It showed better survival in the group treated by VA-ECMO and multivariate analyses identified VA-ECMO as one of the independent factors of survival [24]. Other non-controlled retrospective studies have reported a survival of between 25 and 75% in patients with cardiotoxicant poisonings treated by VA-ECMO [240–245]. A registry study showed improvement of haemodynamic and metabolic parameters of cardiotoxicant-poisoned patients treated by VA-ECMO [246]. A recent study showed that 50% of cardiotoxicant-poisoned patients treated by surgically implanted VA-ECMO developed ischaemia of the cannulated leg *versus* only 16.9% of patients treated by VA-ECMO for cardiac arrest or cardiogenic shock due to other aetiologies [247]. No studies have specifically analysed neurological, haemodynamic or respiratory complications and no studies have specified the indications and criteria for initiation of VA-ECMO therapy, as patients treated by VA-ECMO are generally either in cardiac arrest or present refractory shock. A review of the literature on the management of calcium-channel blocker poisoning found a low level of evidence to support VA-ECMO use [71]. Recommendations for the management of calcium-channel blocker poisoning consider that VA-ECMO can be used in cases of refractory shock in expert centres [72].

## Supplementary information

**Additional file 1.** Classification of the new psychoactive substances (NPS). **Figure S1.** Examples of new psychoactive substances (NPS) mimicking the psychoactive effects of “traditional” molecules. **Figure S2.** The Drugs Wheel, A new model for substance awareness [UK version 2.0.7 dated 08/09/2018—www.thedrugswheel.com.]

## Data Availability

Not applicable.

## Abbreviations

PCC: Poison control centre
ECG: Electrocardiogram
ECMO: Extracorporeal membrane oxygenation
RRT: Renal replacement therapy
ILE: Intravenous lipid emulsion
GC/MS: Gas chromatography linked to mass spectrometry
HRMS: High-resolution mass spectrometry
ER: Extended release
LC/MS/MS: Liquid chromatography linked to tandem mass spectrometry
MDMA: 3,4-Methylenedioxymethamphetamine
MS: Mass spectrometry
NPS: New psychoactive substance
CCU: Continuing Care Unit
VA: Veno-arterial
