# Supplementary material for: Management of pharmaceutical and recreational drug poisoning
Source: Ann Intensive Care. 2020 Nov 23;10:157. doi: 10.1186/s13613-020-00762-9 (PMC7683636; doi:10.1186/s13613-020-00762-9)

**Additional file 1- Classification of the new psychoactive substances (NPS)**

The new psychoactive substances, commonly known as NPS, have been defined by [the United Nations Office on Drugs and Crime](https://www.unodc.org/unodc/fr/) (UNODC) as "*substances of abuse, either in a pure form or a preparation, that are not controlled by the 1961 Single Convention on Narcotic Drugs or the 1971 Convention on Psychotropic Substances, but which may pose a public health threat*". In 2018, a total of about 650 molecules had been identified in Europe and just over 300 molecules had been identified in France, belonging to 11 different chemical families. Several classifications have been proposed. One classification based on chemical structure identifies the following families of substances:

- Aminoindanes
- Arylalkylamines
- Benzodiazepines
- Synthetic cannabinoids
- Cathinones
- Indolalkylamines
- Synthetic opioids
- Phenethylamines
- Piperazines
- Piperidines and pyrrolidines

The chemical structures of these substances may be similar to that of traditional substances, but are sometimes different. NPS are designed to mimic the effects of already known medicinal products or drugs **(Figure 1)** and to circumvent legislation. A classification of NPS based on the desired psychoactive effects compared to traditional psychoactive substances is also proposed (**Figure** **2**).

Many cases of NPS poisoning, involving various substances, have been reported in France over recent years, but only limited published data are available. For example, in the DRAMES (*Décès en relation avec l’abus de médicaments et de substances* [deaths related to drug and substance abuse]) survey set up by the ANSM (Agence nationale de sécurité du médicament et des produits de santé [French Agency for the Safety of Health Products]) – annual prospective study), 36 deaths involving NPS have been reported since 2012. Cathinones (3-MMC, 4-MEC, butylone, MDPV, mephedrone, methylone, mexedrone, penterone, α-PVP) were the substances most often implicated in these deaths in each year of the survey. Other families are also involved: benzofurans (5-APB, 5-APDB, 5-MAPB), arylcyclohexylamines (MXE, MXP), designer benzodiazepines (diclazepam, deschloroetizolam), NBOMe (25C-NBOMe), synthetic opioids (ocfentanil), piperazines (ethylphenidate), other substances (3FPM, MPA).

**Figure S1 -** Examples of new psychoactive substances (NPS) mimicking the psychoactive effects of "traditional" molecules.


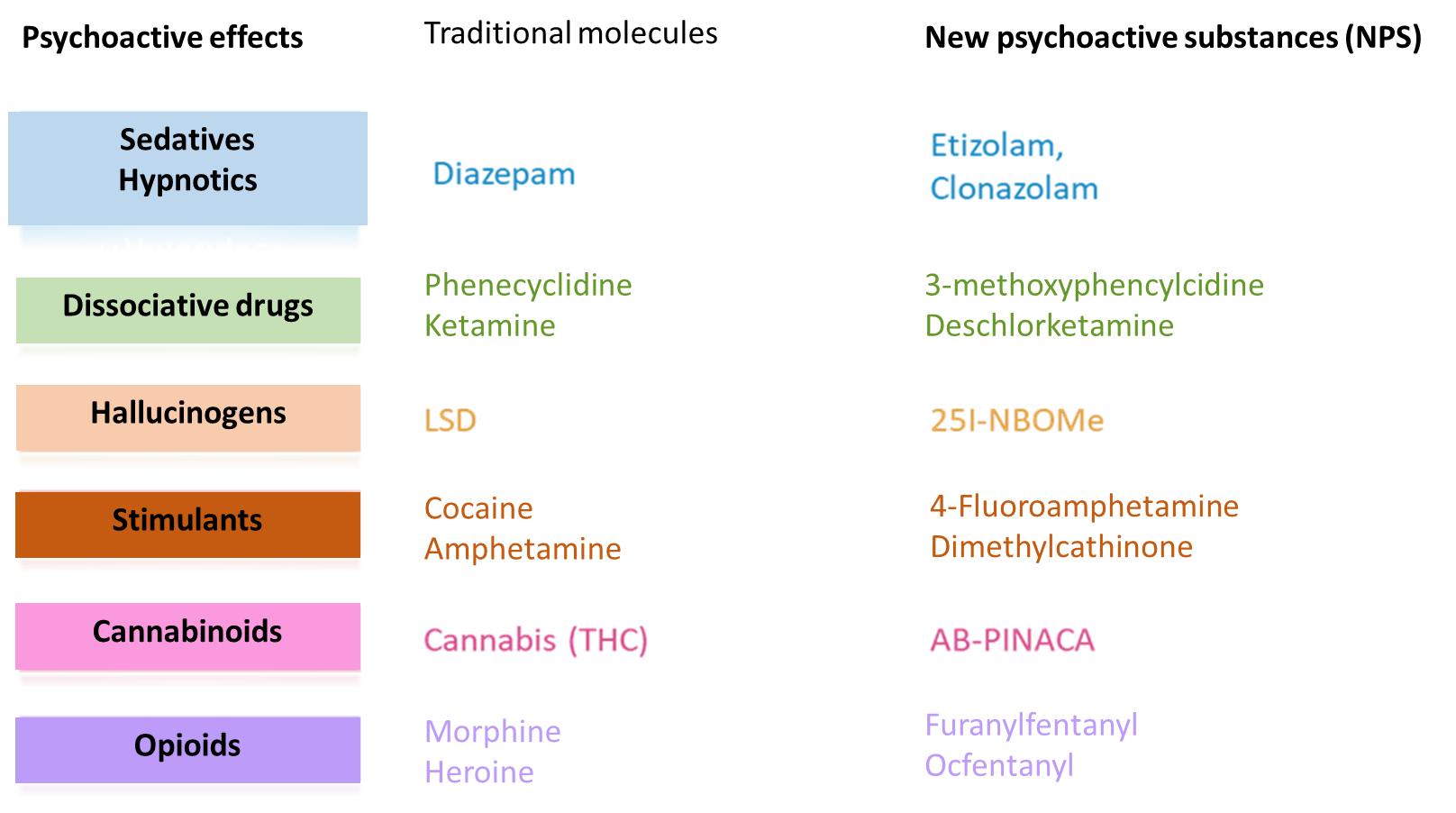


**Figure S2 -** The Drugs Wheel, A new model for substance awareness [UK version 2.0.7 dated 08/09/2018 - [www.thedrugswheel.com](http://www.thedrugswheel.com).]


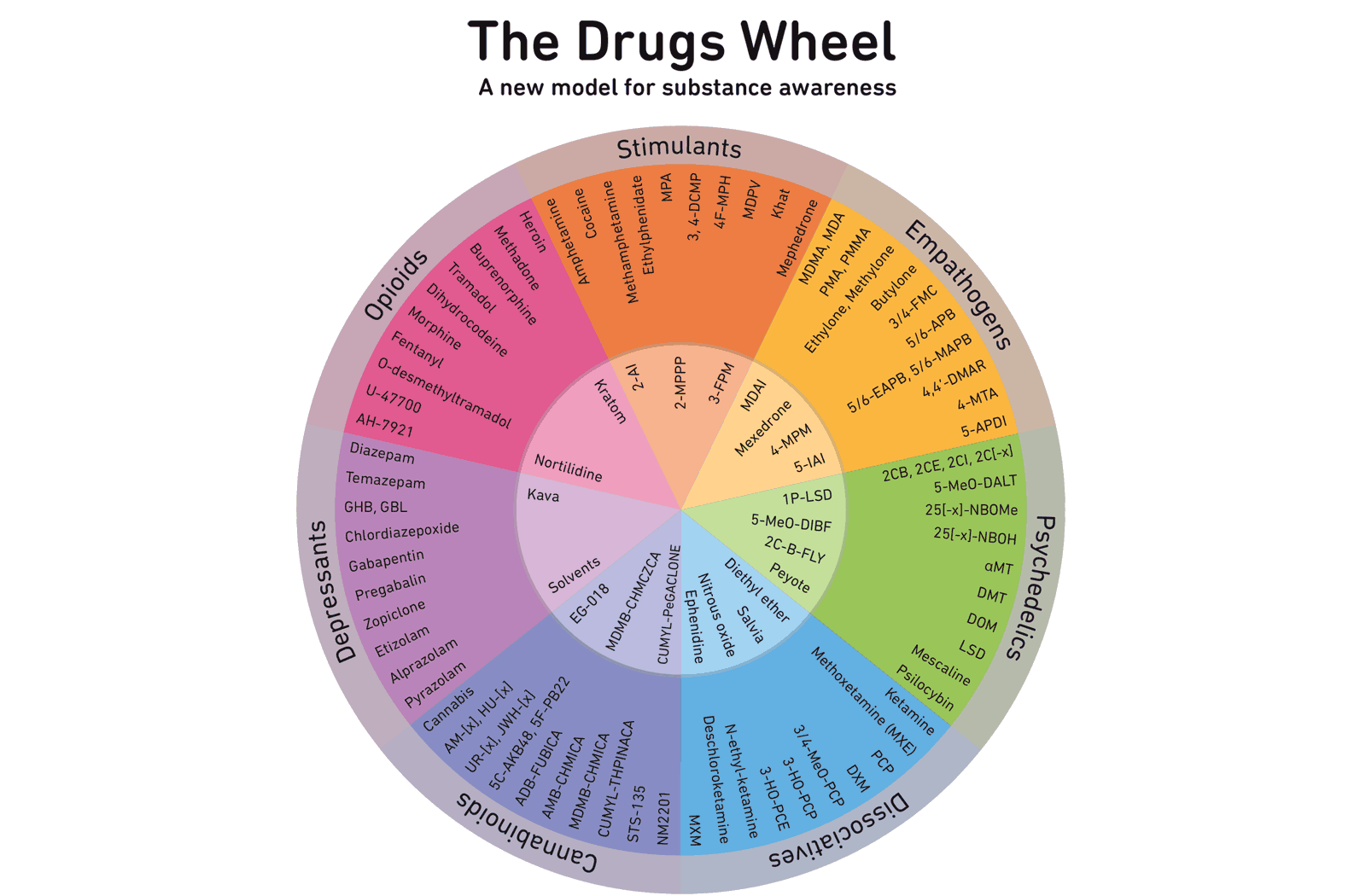

Supplement: Supplementary file 1 — Additional file 1. Classification of the new psychoactive substances (NPS). Figure S1. Examples of new psychoactive substances (NPS) mimicking the psychoactive effects of “traditional” molecules. Figure S2. The Drugs Wheel, A new model for substance awareness [UK version 2.0.7 dated 08/09/2018—www.thedrugswheel.com.] [file 13613_2020_762_MOESM1_ESM.docx]
